# Role of *Aspergillus* neutral protease II in heat-induced soy sauce sediment formation

**DOI:** 10.1007/s00253-026-13923-w

**Published:** 2026-07-03

**Authors:** Takayuki Igarashi, Hideo Ikeuchi, Naho Shinohara, Yuichiro Hashiguchi, Yasuko Araki, Kotaro Ito

**Affiliations:** Research & Development Division, Kikkoman Corporation, 338 Noda, Noda, Chiba 278-0037 Japan

**Keywords:** Heat-induced protein aggregation, Metalloprotease, Protease-deficient mutant, Fermented food biotechnology

## Abstract

**Abstract:**

Soy sauce is produced from fermented soybeans, wheat, and salt by koji molds (*Aspergillus oryzae* and *Aspergillus sojae*) together with salt-tolerant lactic acid bacteria and yeast. Pasteurization, an essential production step, causes protein insolubilization and produces heat-induced sediment that must be removed because it impairs product appearance. Although koji mold–derived proteins have been suggested to contribute to sediment formation, the underlying mechanisms remain unclear. In this study, we aimed to identify the key protein responsible for heat-induced sediment formation. Pasteurizing soy sauce in the presence of a metal chelator prevented sediment formation, and sediment reappeared upon reintroduction of divalent metal ions, indicating that heat-induced aggregation requires metal ion availability. This dependence is consistent with the involvement of neutral protease II (NpII, also known as DeuA or NptB), a thermostable zinc metalloprotease. Soy sauce brewed with *A. oryzae* and *A. sojae* strains deficient in NpII showed no sediment formation. Furthermore, in soy sauce brewed with the NpII-disruptant strains, the addition of a non-thermostable protease preparation after pasteurization restored sediment formation, demonstrating that partial proteolysis occurring only after heat treatment is crucial for sediment generation. Proteomic analysis revealed that > 98% of the proteins in heat-induced sediment originated from koji molds and exhibited highly similar profiles across species and strains. To the best of our knowledge, this is the first study to demonstrate that improvement of koji mold strains can prevent detectable heat-induced sediment formation in soy sauce brewing, contributing to more sustainable manufacturing by eliminating the sediment removal process.

**Key points:**

- *Neutral protease II promotes heat-induced sediment formation in soy sauce.*
- *Heat-induced sediment proteins were > 98% koji mold–derived.*
- *Sediment formation occurs via protease-mediated hydrophobic aggregation.*

**Supplementary Information:**

The online version contains supplementary material available at https://doi.org/10.1007/s00253-026-13923-w.

## Introduction

Soy sauce is a traditional fermented seasoning with a rich umami taste that forms the foundation of “Washoku” (Japanese cuisine). Currently, the annual global production of soy sauce exceeds ten million tons, making it one of the most widely used fermented seasonings worldwide (Ji et al. 2024). In Japan, the most common method of soy sauce production is known as the “honjozo method,” which involves the following steps: a mixture of protein- (such as steamed soybeans) and starch-rich (such as roasted and crushed wheat) ingredients that is inoculated with starter cultures of *Aspergillus oryzae* or *Aspergillus sojae* to produce soy sauce koji. During koji production, various hydrolytic enzymes, such as proteolytic and amylolytic enzymes, are produced by koji molds and are essential for the degradation of the ingredients—soybeans and wheat—into amino acids, peptides, and small sugars. Brine is then added to the koji to create a mash called moromi. Lactic acid fermentation is initiated by the addition of the halophilic lactic acid bacterium *Tetragenococcus halophilus*, followed by inoculation with soy sauce yeast, such as *Zygosaccharomyces rouxii*. Once the pH drops to ~5.2, alcoholic fermentation occurs. In addition to the subsequent aging stage, the fermentation by *Wickerhamomyces versatilis*, which produces key phenolic compounds such as 4-ethylguaiacol, also plays an essential role. After fermentation, moromi is aged for a certain period to facilitate various chemical reactions between its diverse components, such as the Maillard reaction, and then subjected to pressing and filtration to obtain raw soy sauce. Finally, the raw soy sauce is pasteurized, and the heat-induced sediment is removed to produce clear soy sauce (Ito and Matsuyama 2021). Pasteurization is a critical step in soy sauce production that serves to sterilize microorganisms, inactivate residual enzymes, and generate characteristic aromatic compounds via chemical reactions (Ito and Matsuyama 2021; Liang et al. 2019). However, this process also leads to heat-induced sediment formation due to the insolubilization of residual proteins in raw soy sauce (Hashimoto and Yokotsuka 1974a). As sediment negatively affects the visual quality of soy sauce, it is typically removed prior to final product packaging. Although various technologies have been developed to efficiently remove sediment, these methods often require additional equipment and materials (Watanabe et al. 1998; Kadowaki and Noda 2000; Noda and Kadowaki 2000), resulting in process energy demand and additional industrial waste, which can contribute to elevated CO_2_ emissions during production. Recently, the industry has increasingly focused on decarbonization and reducing energy use and waste, which is a key challenge in manufacturing (Naumann et al. 2025). Therefore, developing a fermentation method that prevents sediment formation during pasteurization is important not only for improving yield but also from an environmental sustainability perspective.

Heat-induced sediment in soy sauce comprises 81.6–89.2% protein, 9.6–15.2% carbohydrates, and 1.2–2.3% ash (Hashimoto et al. 1971). The proteins involved in sediment formation are derived from koji mold (Hashimoto et al. 1970a). The addition of koji-derived α-amylase or alkaline protease to raw soy sauce increases sediment formation, whereas the removal of acidic protease reduces sediment formation (Motai et al. 1983). Soy sauce fermented using *A. oryzae* lacking the *amyR*, which regulates the transcriptional activation of α-amylase, has an ~50% reduction in heat-induced sediment (Kitamoto et al. 2008). Hashimoto et al. reported that the addition of alkaline protease, neutral protease I, and neutral protease II from koji molds to heat-treated soy sauce promoted sediment formation. They proposed a model in which neutral protease II, a heat-stable protease, acts on residual proteins during pasteurization at approximately 80 °C, inducing hydrophobic interactions between denatured protein monomers and forming polymers that lead to sediment formation (Hashimoto and Yokotsuka 1974a; b). In contrast, Tamura et al. prepared raw soy sauce from which fractions containing alkaline protease and neutral protease II (molecular weight 10–40 kDa) were removed, and pasteurization tests were conducted after adding purified enzymes. Alkaline proteases promote sediment formation in a dose-dependent manner, whereas neutral protease II does not (Tamura et al. 1987).

Although previous studies have indicated that koji mold–derived proteins are involved in sediment formation, the specific protein components and enzymes responsible for promoting sediment formation remain insufficiently characterized. Mutagenesis-based improvement of koji mold strains has been actively pursued, and in particular, strains with enhanced protease production involved in the degradation of raw material proteins have been successfully developed (Iguchi and Yamamoto 1955; Nasuno et al. 1971). Therefore, in this study, we aimed to identify the enzymes responsible for sediment formation and elucidate their mechanistic roles during pasteurization. Our findings will help develop a more sustainable process for soy sauce production as developing koji molds deficient in sediment-inducing proteins can eliminate heat-induced sediment formation in soy sauce.

## Materials and methods

### Strains and culture conditions

The strains used in this study are listed in Table S1. For the disruption of the neutral protease II gene (*npII*) in *A. sojae* NBRC4239, *A. oryzae* RIB40, and *A. oryzae* RIB326, each Δ*ku70*/Δ*pyrG* (KP-del) strain was used as the host strain. The KP-del strains of *A. sojae* NBRC4239 and *A. oryzae* RIB40 were obtained from previous studies (Araki et al. 2021; Takahashi et al. 2006) and the KP-del strain of *A. oryzae* RIB326 was prepared using the same procedure as *A. oryzae* RIB40. *A. sojae* P6-1 (Araki et al. 2021) was used as a host for the overexpression of *npII* gene.

Strains of *A. sojae* and *A. oryzae* were grown on potato dextrose agar (Nissui Pharmaceutical Co., Tokyo, Japan) for 5 days at 30 °C to harvest conidia. For the preparation of NpII enzyme solution, conidia of *npII*-overexpressing strain of *A. sojae* NBRC4239 (As-npII-OE) were inoculated in the liquid minimal medium consisting of 3.5% Czapek-Dox broth (Becton Dickinson and Co.) and 0.1% trace elements (Cove 1966), adjusted the concentration to 1 × 10^5^ conidia/mL, and cultivated for 7 days at 30 °C on a rotary shaker at 180 rpm.

### Transformant construction for functional NpII analysis in *A. sojae* and *A. oryzae*

The primers used for strain construction are listed in Table S2. The DNA fragments used to construct the transformants were amplified using KOD One PCR Master Mix (Toyobo, Osaka, Japan) and fused using an In-Fusion HD Cloning Kit (Clontech Laboratories, Mountain View, CA, USA). Transformation of *A. sojae* and *A. oryzae* was performed using the protoplast-polyethylene glycol method, as previously reported (Araki et al. 2019). The *npII* gene in *A. sojae* was identified as an ortholog of gene AO090010000493 in *A. oryzae* using *A. sojae* NBRC4239 genome data (Sato et al. 2011).

To disrupt the *npII* gene of *A. sojae* NBRC4239, *A. oryzae* RIB40, and* A. oryzae* RIB326, each deletion cassette containing *pyrG* marker between 5′-upstream and 3′-downstream regions of *npII* gene was constructed. The DNA fragments were amplified from the genomic DNA of *A. sojae* NBRC4239, *A. oryzae* RIB40, and *A. oryzae* RIB326 using the primers listed in Table S2 and assembled into the linearized pUC19 vector using an In-Fusion HD Cloning Kit. The KP-del strains of *A. sojae* NBRC4239, *A. oryzae* RIB40, and *A. oryzae* RIB326 were transformed with each deletion cassette, and the Δ*npII* strains in which the region containing the full-length *npII* ORF has been replaced with the *pyrG* marker were obtained. Control strains (K-del) were obtained by complementing *pyrG* gene at its original locus with the KP-del strains of *A. sojae* NBRC4239, *A. oryzae* RIB40, and *A. oryzae* RIB326.

To obtain the *npII*-overexpressing strain of *A. sojae* NBRC4239 (As-npII-OE), a multi-copy integrative vector (pUC19-pG56-npII) was constructed by replacing *Mpgdh* with *npII* under the control of the translation elongation factor promoter (P*tef*) in a vector containing the attenuated *pyrG* marker (*pyrG56*) previously described (Araki et al. 2021). To construct pUC19-pG56-npII, the *npII* gene was amplified from the genomic DNA of *A. sojae* NBRC4239 and inserted into a linearized multi-copy integrative vector obtained by inverse PCR using the primers listed in Table S2. The *A. sojae* P6-1 strain was transformed with pUC19-pG56-npII, and a high-copy strain was selected by over-amplification of the on-plasmid amplicon compared to the endogenous amplicon in a multiplex PCR setting, and then by overexpression of *npII* in sodium dodecyl sulfate–polyacrylamide gel electrophoresis (SDS-PAGE) analysis.

To obtain the complemented strain (As-npII-WT_CP) in *A. sojae* Δ*npII*, DNA fragments of the *npII* expression cassette containing promoter (1127 bp) and terminator (1000 bp) of *npII* gene from *A. sojae*, the pyrithiamine resistance gene (*ptrA*), and the vector containing the 5′-upstream and 3′-downstream regions of the GDH-like gene from *A. sojae* (*Asgdh*; ortholog of AO090103000214, protein sequence identity 95%) were amplified from genomic DNA of *A. sojae* NBRC4239, pPTR1 (Takara Bio Inc., Shiga, Japan) and the vector previously constructed (Araki et al. 2021), using primers listed in Table S2. The plasmid pUC19-npII-WT-CP for *A. sojae* Δ*npII* complement was obtained by fusing the above each DNA fragment using an In-Fusion HD Cloning Kit. To construct a plasmid containing the expression cassette of *npII* with a nonsense mutation (Fig. S1), pUC19-npII-WT-CP was used as the template. Site-directed mutagenesis was performed using a KOD-Plus-Mutagenesis Kit (Toyobo) to generate pUC19-npII-mut1-CP with npII-mut1-Fw and npII-mut1-Rv, and pUC19-npII-mut2-CP with npII-mut2-Fw and npII-mut2-Rv. The complemented strains of *A. sojae* Δ*npII* (As-npII-WT_CP, As-npII-mut1_CP, As-npII-mut2_CP) were obtained by transformation with the DNA fragment amplified from pUC19-npII-WT-CP, pUC19-npII-mut1-CP, and pUC19-npII-mut2-CP using primers, GDH-5arm-Fw and GDH-3arm-Rv.

### Koji and soy sauce production

Koji and soy sauce were prepared as previously described (Ito et al. 2013). Briefly, equal amounts of steamed soybeans and roasted wheat were mixed and inoculated with the koji starters of the control (K-del) and *npII*-disruptant strains. The mixture was cultured for 3 days under a controlled temperature of 20–30 °C at 95% humidity, resulting in a dry mash (the koji). Koji was mixed with concentrated saline water (brine, 120–130% of the ingredient volume) and added to a pure culture of *T. halophilus*. As fermentation progressed and the pH dropped to ~5.2 (after approximately 1–2 months), pure culture of *Z. rouxii* was added. The moromi was stored at 15–30 °C for 4 months with occasional aeration, followed by anaerobic storage for alcohol fermentation. Raw soy sauce was obtained by filtering moromi through a filter paper, followed by additional filtration using diatomaceous earth.

### Enzyme activity assays

Koji extract was prepared for enzyme activity assay as follows: 100 mL Milli-Q (MQ) water was added to 10 g koji. The mixture was then stirred hourly at room temperature (~25 °C) for 3 h. After stirring, the mixture was filtered through a No. 2 filter paper (ADVANTEC, Tokyo, Japan) to obtain the koji extract, which was used for enzyme activity assays.

Alkaline protease activity was measured using casein as a substrate (Cupp-Enyard. 2008). A 2% milk casein solution was prepared in 0.2 M phosphate buffer (pH 7.0) as the substrate. A reaction mixture containing 50 µL of koji extract, 450 µL of MQ water, and 500 µL of substrate solution was incubated at 30 °C for 30 min. The reaction was stopped by adding 2 mL of 5% trichloroacetic acid (TCA), followed by incubation at 30 °C for 30 min. A blank was prepared by adding TCA prior to starting the reaction. After filtration through No. 5 filter paper (ADVANTEC), 500 µL of the filtrate was mixed with 2.5 mL of 0.4 M sodium carbonate (FUJIFILM Wako Pure Chemical Corporation, Osaka, Japan) and 500 µL of 20% Folin & Ciocalteu’s phenol reagent (MP Biomedicals, Irvine, CA, USA), and incubated at 30 °C for 30 min. Absorbance was measured at 660 nm using a U-3900 UV-Vis spectrophotometer (Hitachi High-Tech Corporation, Tokyo, Japan). A protease from *A. oryzae* (Sigma-Aldrich, St. Louis, MO, USA) was used as a standard to correct the measured values. One unit (U) of activity was defined as the amount of enzyme that releases 1 μg of tyrosine equivalents from milk casein per min at 30 °C.

Leucine aminopeptidase activity was measured using 1 mM _L_-Leucyl-p-nitroanilide hydrochloride (FUJIFILM Wako Pure Chemical Corporation) as a substrate (Matsushita-Morita et al. 2011). A reaction mixture containing 292.5 µL of substrate solution and 7.5 µL of koji extract was incubated at 30 °C for 40 min. The reaction was stopped by adding 900 µL of 0.1 N HCl, and absorbance was measured at 400 nm using a U-3900 UV-Vis spectrophotometer (Hitachi High-Tech Corporation, Tokyo, Japan). One unit (U) of activity was defined as the amount of enzyme that releases 1 μmol of p-nitroanilide from _L_-Leucyl-p-nitroanilide per min at 30 °C.

Acid carboxypeptidase activity and α-amylase activity were measured using acid carboxypeptidase and α-amylase assay kits (both from Kikkoman Biochemifa Co., Ltd., Tokyo, Japan), respectively.

Neutral protease II activity was measured using Boc-Arg-Val-Arg-Arg-MCA (Peptide Institute, Inc., Osaka, Japan) as a substrate (Doi et al. 2003). A 10 µL aliquot of enzyme solution, heat-treated at 80 °C for 10 min, was added to 90 µL of 200 µM substrate solution (50 mM Tris-HCl, pH 7.0). The reaction mixture was incubated at 50 °C for 2.5, 5, 7.5, or 10 min. The reaction was stopped by adding 200 µL of 0.1 M sodium acetate buffer (pH 4.5). The fluorescence intensities of the reaction mixtures and AMC standard solutions (0, 50, 100, and 150 pmol/well; Tokyo Chemical Industry Co., Ltd., Tokyo, Japan) were measured using an Infinite 200 microplate reader (Tecan Group Ltd., Männedorf, Switzerland) at excitation and emission wavelengths of 375 and 450 nm, respectively. A calibration curve was constructed from the AMC standards, and one unit (U) of enzyme activity was defined as the amount of enzyme that produces 1 µmol of AMC/min at 50 °C.

### Chemical component analysis of soy sauce

The total nitrogen concentration in the soy sauce was measured by the combustion method using a Dumatherm N Pro analyzer (C. Gerhardt GmbH & Co. KG, Königswinter, Germany).

The amino acid composition was analyzed using a Type H amino acid standard solution (FUJIFILM Wako Pure Chemical Corporation) with a Hitachi high-speed amino acid analyzer L-8900 (Hitachi High-Tech Corporation, Tokyo, Japan).

The ethanol concentration in soy sauce was quantified using gas chromatography with flame ionization detection (GC/FID) using a GC-2014 AF system (Shimadzu Corporation, Kyoto, Japan). The lactic acid concentration was measured using the immobilized enzyme electrode of the biosensor BF-9 (Able Co., Tokyo, Japan).

### Evaluation of heat-induced sediment formation

Pasteurization was performed by heating the raw soy sauce at 80 °C for 30 min. After heating, the soy sauce was transferred to a 20-mL graduated cylinder and incubated at 57 °C for 72 h, followed by overnight incubation at 16 °C to promote sedimentation. The volume of settled sediment was measured.

To evaluate the turbidity generated after heat treatment, the supernatant turbidity and total turbidity (after thoroughly mixing with the sediment) of the soy sauce were measured. Turbidity was measured at 660 nm using a UT-21 turbidimeter (Corona Electric Co., Ltd., Ibaraki, Japan). In this study, turbidity was recorded as the value directly displayed by the UT-21. The UT-21 determines turbidity based on the ratio of forward-scattered light to transmitted light, and the instrument automatically converts the signal into turbidity units (ppm), where 1 ppm corresponds to the turbidity produced by 1 mg of kaolin per liter of water, according to the manufacturer’s specification.

A supplementation assay using EDTA and metal ions was performed as follows: raw soy sauce was purchased from the Kikkoman Corporation (Tokyo, Japan). This raw soy sauce was heated at 80 °C for 30 min. After heating, a 500 mM EDTA solution (pH 8.0) (Nippon Gene, Ltd., Tokyo, Japan) was added to the sample to achieve a final concentration of 5 mM EDTA. Each metal ion was prepared as a 1 M solution and added to achieve a final concentration of 10 mM. The samples were incubated to promote sedimentation as described above.

A supplementation assay for the NpII enzyme solution was performed as follows: the culture broth of As-npII-OE was first filtered through No. 2 filter paper (ADVANTEC), followed by membrane filtration using a 0.45-µm filter (ADVANTEC) to obtain the enzyme solution. The enzyme solution was then concentrated using a 10 K Amicon ultrafiltration membrane (Merck KGaA, Darmstadt, Germany) to adjust the enzyme activity to 125 mU/mL. This NpII enzyme solution was added to raw soy sauce produced using the *npII*-disruptant strain to a final concentration of 5 mU/mL with or without 5 mM EDTA. When the NpII enzyme solution was added after heat treatment, the raw soy sauce was first subjected to heat treatment in the presence of 0.1% protease inhibitor cocktail III (EDTA-free) (FUJIFILM Wako Pure Chemical Corporation, Osaka, Japan) and 5 mM EDTA. After heat treatment, NpII enzyme solution was added to a final concentration of 5 mU/mL, along with ZnSO_4_·7H_2_O (FUJIFILM Wako Pure Chemical Corporation, Osaka, Japan) to a final concentration of 10 mM.

For the protease supplementation assay, final concentrations of 0.1% papain (FUJIFILM Wako Pure Chemical Corporation), 0.1% protease from *Streptomyces griseus* (Sigma-Aldrich), and 0.1% protease from *Bacillus* sp. (Sigma-Aldrich) were added to the heat-treated soy sauce produced using the *npII*-disruptant strain. For preparation of the heat-inactivated protease, 10% protease enzyme solutions were pre-treated by heating at 100 °C for 20 min to ensure thermal inactivation.

### Proteomic analysis of raw soy sauce and heat-induced sediment

Proteomic analysis was performed on raw soy sauce and heat-induced sediment prepared using the control strains (K-del) of *A. oryzae* RIB40 and RIB326, and *A. sojae* NBRC4239. The heat-induced sediment samples were pre-treated by centrifuging the sediment-containing fraction at 20,379× *g* for 10 min, followed by three washes with phosphate-buffered saline PBS (FUJIFILM Wako Pure Chemical Corporation).

Proteomic analysis was outsourced to Promega Corporation (Madison, WI, USA) and performed using data-independent acquisition (DIA) on an Orbitrap Exploris 480 mass spectrometer (Thermo Fisher Scientific, Waltham, MA, USA). Label-free quantification (LFQ) data were analyzed using DIA-NN version 2.2.0 Enterprise.

To construct the predicted spectral library, reference sequences were retrieved from public databases, including the National Center for Biotechnology Information (NCBI; https://www.ncbi.nlm.nih.gov/) and UniProt (https://www.uniprot.org). For the *A. sojae* SMF134, which lacked annotated protein predictions, the genome sequence was obtained from the NCBI, and gene annotation was performed using Helixer version 0.3.4 in lineage-specific mode with “Fungi” selected. Similarly, the genome sequence of *Z. rouxii* NBRC1876 was retrieved from NCBI and annotated using Augustus on the Galaxy platform (https://usegalaxy.org) with the gene model set for *Saccharomyces cerevisiae* strain S288C.

## Results

### Effects of metal ions on heat-induced sediment formation

**Fig. 1:**
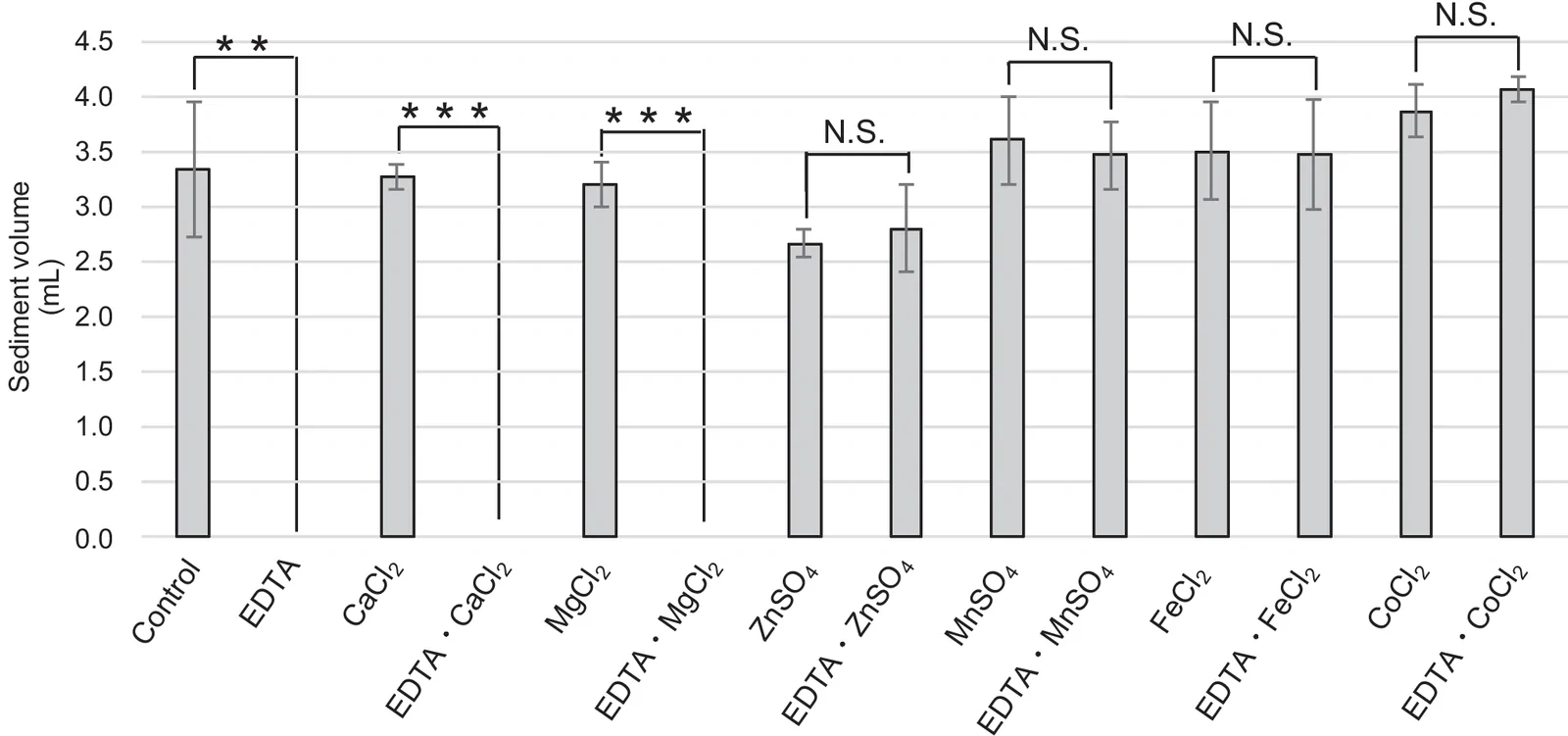
Evaluation of the effects of EDTA and metal ions on heat-induced sediment formation. Raw soy sauce (20 mL) was heat-treated at 80 °C for 30 min and then supplemented with 5-mM EDTA and/or 10-mM metal ions, followed by incubation at 57 °C for 72 h to promote sedimentation. The volume of sediment was measured after the sample was allowed to stand overnight at 16 °C. Data are presented as the means of three independent experiments. Error bars indicate standard deviation. Statistical significance was evaluated by Welch’s t-test. Asterisks indicate statistically signifi cant differences (*p <0.05, **p < 0.01, ***p < 0.001), and N.S. indicates no significant difference

The metal ion–chelating agent, EDTA, suppresses heat-induced sediment formation (Hashimoto et al. 1970b). To investigate the factors involved in sediment formation, we examined whether the addition of EDTA and excess amounts of various metal ions to soy sauce induced heat-induced sediment formation (Fig. 1). As previously reported, EDTA effectively suppressed heat-induced sediment formation. The addition of excess Ca^2^⁺ or Mg^2^⁺ did not suppress the effects of EDTA. In contrast, the addition of Zn^2^⁺, Mn^2^⁺, Fe^2^⁺, or Co^2^⁺ counteracted the effect of EDTA and promoted heat-induced sediment formation. These results suggest that several divalent metal ions are involved in heat-induced sediment formation.

### Brewing soy sauce using npII-disruptant strains

**Fig. 2:**
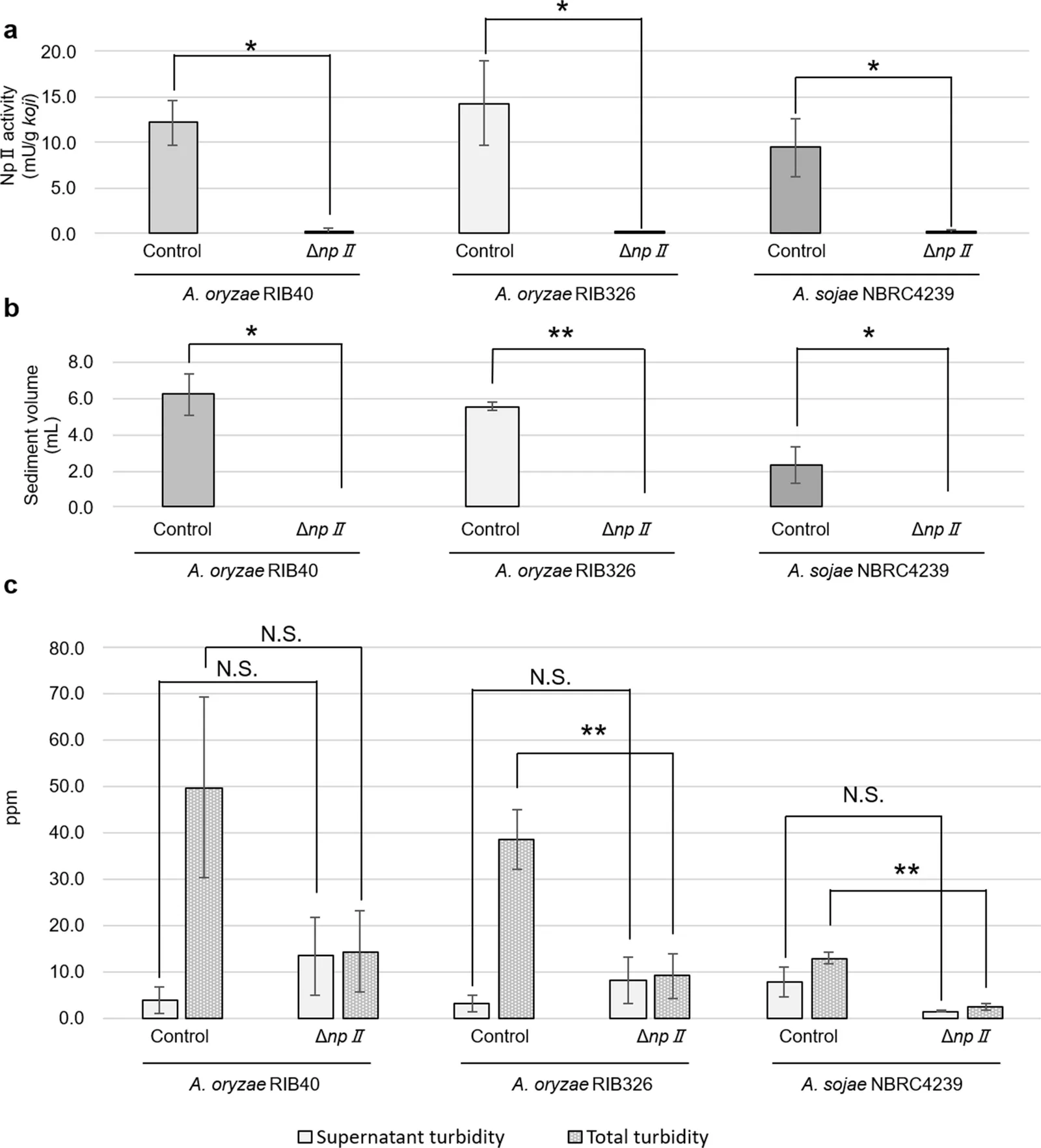
Evaluation of koji and soy sauce using *npII*-disruptant strains. **a** NpII activity in the produced koji. **b** Volume of the heat-induced sediment in the produced soy sauce. **c** Turbidity of the soy sauce produced by control strains and Δ*npII* strains. Data represent the mean ± SD of three independent experiments (*n* = 3). Statistical significance was evaluated by Welch’s t-test. Asterisks indicate statistically signifi cant differences (*p <0.05, **p < 0.01, ***p < 0.001), and N.S. indicates no significant difference

To examine the involvement of neutral protease II (NpII) in heat-induced sediment formation, soy sauce brewing was performed using npII-disrupted strains (Δ*npII*). The Δ*npII* strains of *A. oryzae* RIB40, *A. oryzae* RIB326, and *A. sojae* NBRC4239 were constructed, and soy sauce koji were prepared using these strains. Enzymatic activity assays of the koji revealed that only the activity of NpII was abolished, while the activities of other major proteases and α-amylase were retained (Fig. 2a, Fig. S2). Soy sauce was produced using koji, and heat-induced sediment formation was evaluated. Notably, no detectable heat-induced sediment was detected in the soy sauce produced using the Δ*npII* strains (Fig. 2b). Furthermore, in *A. oryzae* RIB326 and *A. sojae* NBRC4239, a significant reduction of total turbidity was observed in the Δ*npII* strains compared with the control strains (Fig. 2c). In *A. oryzae* strain RIB40, a tendency toward reduced total turbidity was also observed in the Δ*npII* strain compared with the control strain (Fig. 2c). In addition, the complemented Δ*npII* strains of the *A. sojae* NBRC4239 (As-npII-WT_CP, As-npII-mut1_CP, and As-npII-mut2_CP) were constructed and soy sauce brewing was performed using these complemented strains. Heat-induced sedimentation was observed in the strain complemented with the wild-type *npII* gene (As-npII-WT_CP), whereas no sedimentation was detected in the strains complemented with a nonsense-mutated *npII* gene (As-npII-mut1_CP and As-npII-mut2_CP) (Fig. S3). These results indicate that NpII is a key promoter of sediment formation during pasteurization.

To further evaluate the quality of soy sauce fermented with each strain, the total nitrogen and amino acid concentrations were analyzed (Fig. S4a, Fig. S5). The results showed no significant differences between the Δ*npII* strains and their respective control strains in terms of the major components of soy sauce. These results suggest that utilizing *npII*-disruptant strains suppresses heat-induced sediment formation without significantly altering the major compositional characteristics of soy sauce.

### Functional NpII analysis in heat-induced sediment formation

**Fig. 3:**
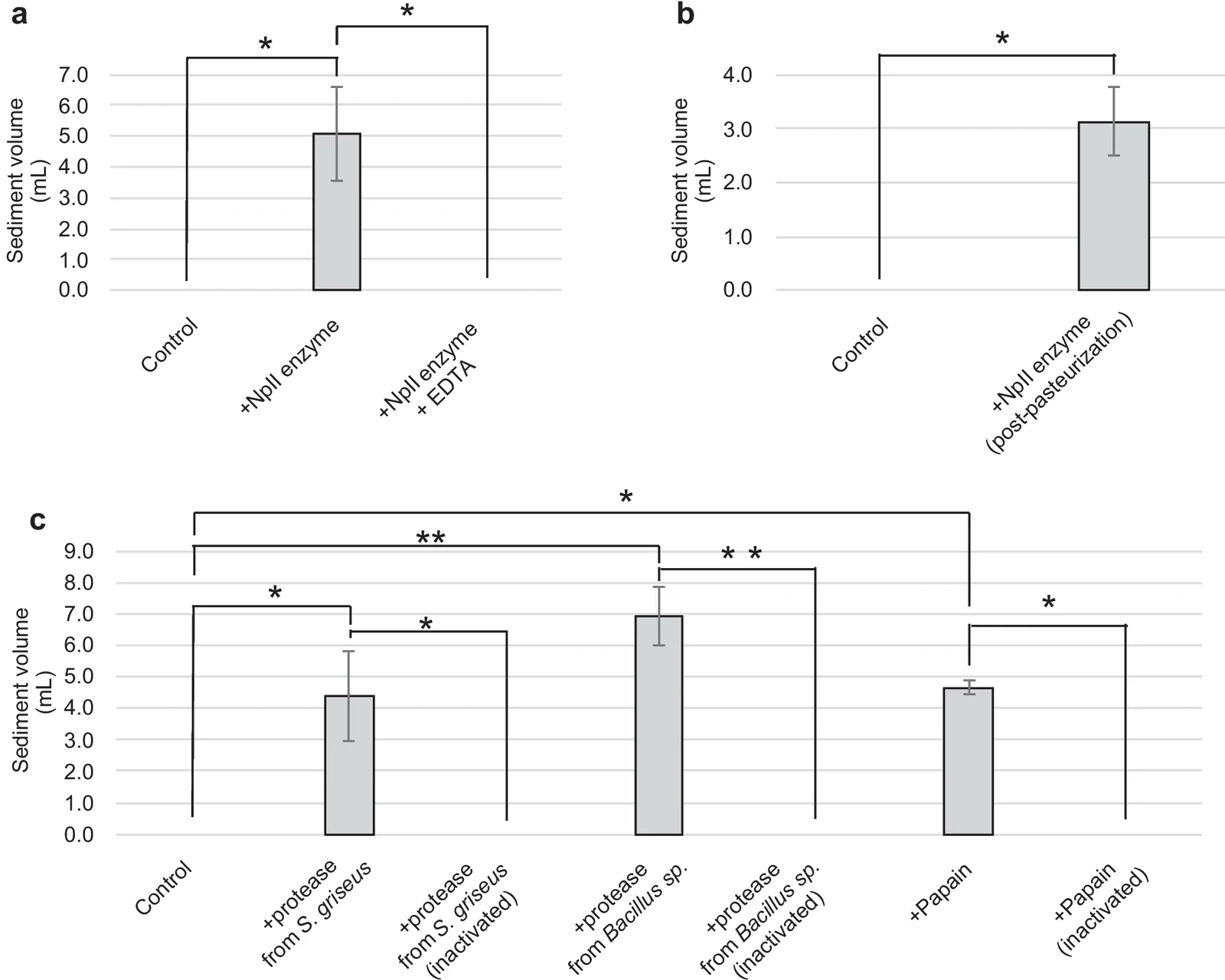
Evaluation of heat-induced sediment formation in raw soy sauce produced using *npII*-disruptant strains after protease addition. **a** Sediment volume after addition of NpII enzyme solution with or without EDTA prior to pasteurization. **b** Sediment volume after complete suppression of protease activity during pasteurization by addition of protease inhibitor cocktail III (EDTA-free) together with 5-mM EDTA, followed by post-pasteurization supplementation with NpII enzyme solution and excess of ZnSO_4_·7H_2_O to restore NpII activity. Protease inhibitor cocktail III (EDTA-free) inhibits serine, cysteine, and aspartic proteases but not metalloproteases. **c** Sediment volume after addition of various protease preparations to heat-treated soy sauce. Proteases were inactivated by heating at 100℃ for 20 min. Data are presented as the means ± SD of three independent experiments (*n* = 3). Statistical significance was evaluated by Welch’s t-test. Asterisks indicate statistically signifi cant differences (*p <0.05, **p < 0.01, ***p < 0.001), and N.S. indicates no significant difference

To investigate how NpII promotes heat-induced sediment formation, an NpII supplementation assay was performed. An NpII enzyme solution was prepared in which NpII was confirmed as a single band by SDS-PAGE (Fig. S6 lane 3). The NpII enzyme solution with or without EDTA was added to the raw soy sauce produced using Δ*npII* strains, and the amount of heat-induced sediment was evaluated. Consequently, the addition of NpII enzyme led to sediment formation when EDTA was absent, whereas no sediment formation was observed when EDTA was present (Fig. 3a). In addition, when the NpII enzyme solution and an excess amount of Zn^2^⁺ were added to the soy sauce that had been heat-treated in the presence of a protease inhibitor containing EDTA, heat-induced sediment formation was observed (Fig. 3b). These results indicate that heat-induced sediment formation is triggered by NpII-mediated degradation of heat-denatured proteins.

Moreover, heat-induced sediment formed when protease preparations other than NpII were added to raw soy sauce after heat treatment, whereas no sediment was observed when heat-inactivated protease preparations were added (Fig. 3c). These results suggest that the exceptional thermal stability of NpII is a critical factor contributing to sediment formation and that sediment can be formed through the reaction of heat-denatured proteins with other proteases that remain active after pasteurization.

### Proteomic analysis of raw soy sauce and heat-induced sediment

To investigate the protein composition of the heat-induced sediment, proteomic analysis of raw soy sauce and heat-induced sediment produced using each control strain was performed. From raw soy sauce and heat-induced sediment produced using *A. oryzae* RIB40, 2,150, and 953 peptides; from *A. oryzae* RIB326, 1,539, and 441 peptides; and from *A. sojae* NBRC4239, 1,561, and 745 peptides were detected. The relative abundance of proteins derived from different organisms was determined based on the summed signal intensities of the unique peptides (Table 1). Proteins derived from koji molds accounted for the largest proportion in both raw soy sauce and heat-induced sediments. In the heat-induced sediment, > 98% of the detected proteins were derived from koji molds. These results indicate that the heat-induced soy sauce sediment is primarily composed of several hundred or more proteins produced by koji molds, with minimal contributions from proteins derived from ingredients (soybeans and wheat) or other fermentation microorganisms, such as soy sauce yeast and lactic acid bacteria.

**Table 1:** Proteomic analysis of the produced raw soy sauce and heat-induced sediment using control strains

Species	Sum of peptide log10 intensities (arbitrary units)						Proportion of total peptide intensity (%)					
	*A. oryzae* RIB40		*A. oryzae* RIB326		*A. sojae* NBRC4239		*A. oryzae* RIB40		*A. oryzae* RIB326		*A. sojae* NBRC4239	
	Raw soy sauce	Sediment	Raw soy sauce	Sediment	Raw soy sauce	Sediment	Raw soy sauce	Sediment	Raw soy sauce	Sediment	Raw soy sauce	Sediment
*Aspergillus oryzae*/*Aspergillus sojae*	12.03	12.07	12.09	12.13	12.05	12.15	89.10	98.76	90.73	99.60	79.48	99.48
*Zygosaccharomyces rouxii*	10.05	9.21	9.45	8.99	9.68	8.96	0.93	0.14	0.21	0.07	0.34	0.06
*Tetragenococcus halophilus*	10.90	10.09	10.97	9.03	11.39	9.66	6.69	1.03	6.88	0.08	17.41	0.32
*Glycine max*	10.56	8.92	10.47	9.48	10.58	9.26	3.09	0.07	2.15	0.22	2.70	0.13
*Triticum aestivum*	9.35	7.76	8.54	8.55	9.00	8.10	0.19	0.00	0.03	0.03	0.07	0.01

**Fig. 4:**
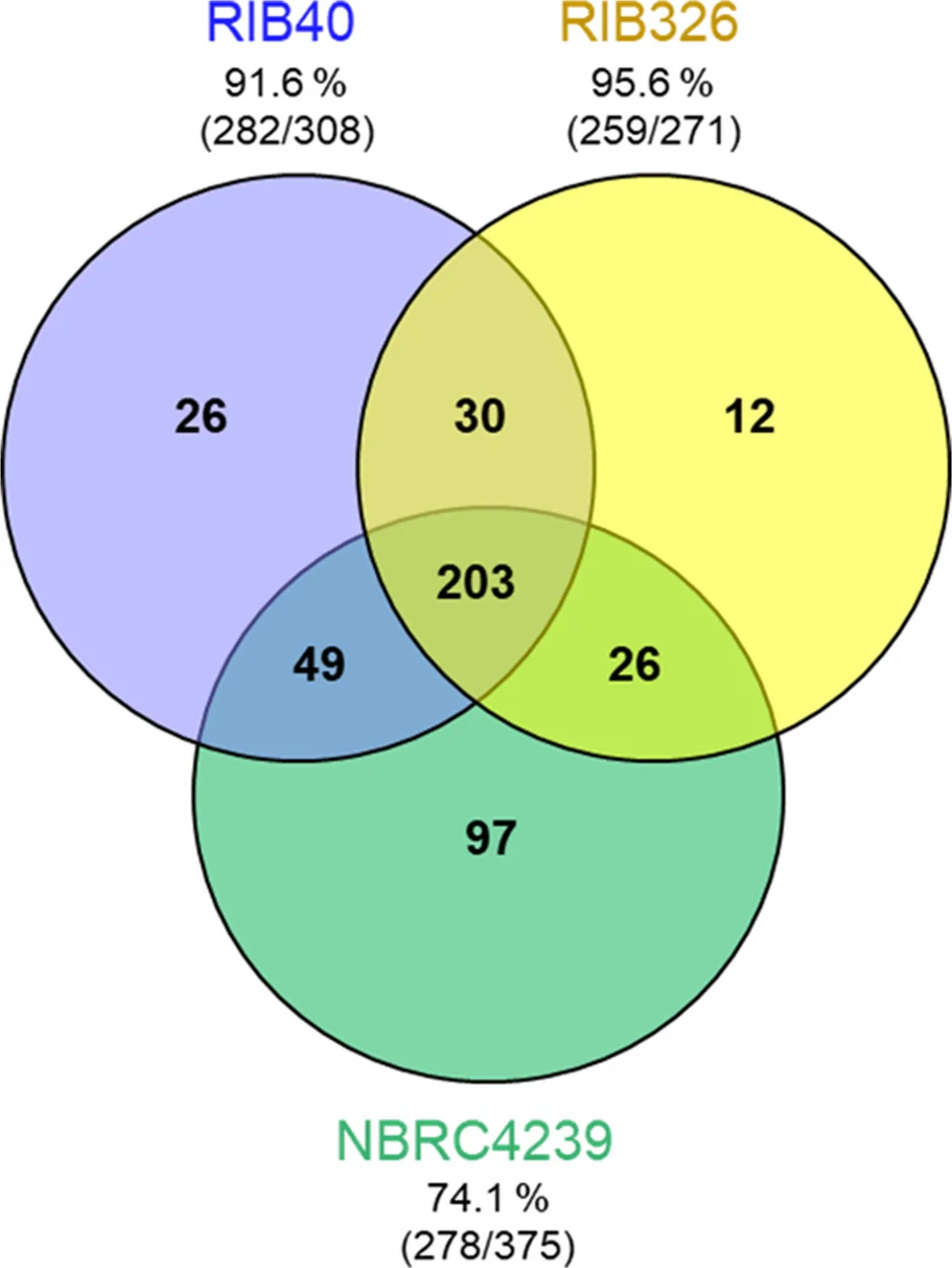
Venn diagrams of koji mold–derived proteins detected in heat-induced sediment. The percentages indicate the proportion of proteins that were commonly detected between each strain and the other two strains

The shared koji mold–derived proteins detected in the heat-induced soy sauce sediment produced by each strain are represented by Venn diagrams (Fig. 4). The proportion of proteins detected in each strain that was also identified in the other two strains was remarkably high: 91.6% for *A. oryzae* RIB40, 95.6% for *A. oryzae* RIB326, and 74.1% for *A. sojae* NBRC4239. These findings indicate that proteins that form heat-induced sediments are commonly present, regardless of the species or strain of koji mold.

**Fig. 5:**
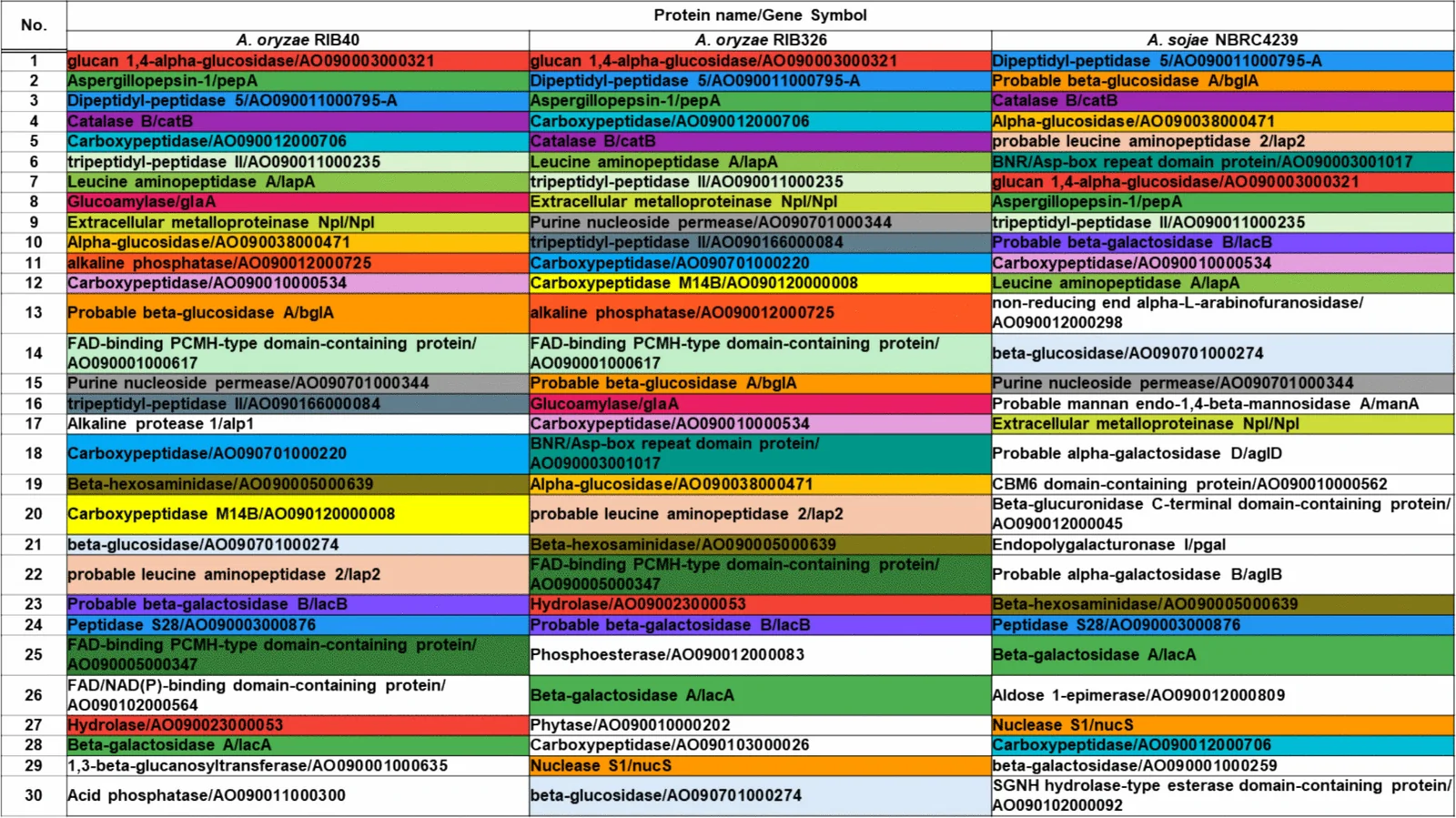
Top 30 proteins detected in heat-induced sediment. Proteins were ranked based on normalized label-free quantification (LFQ) values obtained by DIA-MS analysis, calculated as the sum of unique peptide intensities divided by protein molecular weight. Gene symbols of *A. sojae* were annotated based on their orthologs in *A. oryzae*. The same color indicates orthologous proteins

The top 30 proteins with the highest signal intensities in raw soy sauce and heat-induced sediment are presented (Fig. 5 and Fig. S7‐S9). Numerous extracellular enzymes involved in soy sauce brewing, such as proteases, amylases, and plant cell wall-degrading enzymes, have been detected in both raw soy sauce and heat-treated sediment. These findings suggest that proteins secreted by koji molds, which play important roles in soy sauce brewing, persist until just before heat treatment and subsequently precipitate as a sediment following partial degradation by NpII.

## Discussion

Although enzymes such as NpII, alkaline protease, and α-amylase produced by koji mold (*A. oryzae* or *A. sojae*) have been suggested to be involved in heat-induced sediment formation, the specific enzymes primarily responsible for sedimentation remain unknown. Neutral protease II (NpII, also known as DeuA or NptB), produced by *Aspergillus* species, is a thermostable metalloprotease that remains stable even at temperatures approaching 100 °C and utilizes metal ions such as Zn^2^⁺, Co^2^⁺, and Mn^2^⁺ as cofactors (Maeda et al. 2016; Sekine 1972, 1973; Tatsumi et al. 1991). In this study, we confirmed that heat-induced sediments in soy sauce were eliminated by the addition of EDTA, as previously reported (Hashimoto et al. 1970b). Furthermore, we found that Zn^2^⁺, Mn^2^⁺, Co^2^⁺, and Fe^2^⁺ counteract the suppressive effect of EDTA on heat-induced sediment formation and instead promote sediment generation. The reason for sediment formation upon the addition of Zn^2^⁺, Mn^2^⁺, and Co^2^⁺ is that NpII, which had lost its activity due to EDTA, was reactivated through binding with these metal ions. In contrast, the NpII apoenzyme does not utilize Fe^2^⁺ as a cofactor (Sekine 1972). Nevertheless, the addition of Fe^2^⁺ led to heat-induced sediment formation, which may be attributed to the oxidation of Fe^2^⁺ to Fe^3^⁺ in soy sauce, resulting in the formation of a highly stable complex with EDTA. Since Fe^3^⁺ has a much higher chelation constant than Zn^2^⁺, which is bound to NpII, it is conceivable that a ligand exchange reaction occurred between EDTA-Zn^2^⁺ and Fe^3^⁺, allowing the NpII apoenzyme to rebind Zn^2^⁺ and regain its enzymatic activity.

No heat-induced sediments were formed when soy sauce was produced using the *npII*-disruptant strains (Fig. 2). Furthermore, when the NpII enzyme solution was added to soy sauce produced using the *npII*-disruptant strains, sediment formation was confirmed. Moreover, the addition of various proteases to heat-treated soy sauce resulted in sediment formation (Fig. 3). These findings clearly indicate that the residual protease activity after heat treatment plays a crucial role in heat-induced sediment formation. Conversely, previous studies have reported that heat-labile alkaline proteases are the major factors that promote heat-induced sediment formation (Tamura et al. 1987). As shown in Fig. 3a, heat-induced sediment was formed even when a protease inhibitor was present during heat treatment, suggesting that alkaline protease activity during heating is not required for sediment formation. Furthermore, Tamura et al. reported that the addition of heat-denatured and inactivated alkaline proteases promotes sediment formation. One possible explanation is that heat-denatured alkaline proteases may be degraded by NpII and subsequently exhibit adhesive-like properties, strongly attracting the precursor substances of the heat-induced sediment. However, the roles of alkaline proteases in sediment formation require further investigation.

The main component of the heat-induced sediment was presumed to be protein derived from koji molds. In this study, proteomic analysis of soy sauce sediments produced using three different strains revealed that over 98% of the proteins in the heat-induced sediment originated from koji mold in all samples (Table 1). The protein group found in heat-induced sediment is derived from koji molds based on indirect evidence, such as amino acid analysis of heat-induced sediment and antigen-antibody reactions targeting the sediment (Hashimoto et al. 1970a; Hashimoto and Yokotsuka 1976). To the best of our knowledge, this is the first study to directly demonstrate that the proteins found in heat-induced sediments originate from koji molds and comprise several hundred distinct proteins. The koji mold–derived proteins detected in the heat-induced sediment exhibited a high degree of commonality, with 91.6% for *A. oryzae* RIB40, 95.6% for *A. oryzae* RIB326, and 74.1% for *A. sojae* NBRC4239, compared to the other two strains (Fig. 4). *A. sojae* NBRC4239 exhibited a slightly lower commonality than the two *A. oryzae* strains. The major proteins detected in both raw soy sauce and heat-induced sediment were extracellular enzymes that contribute to soy sauce brewing, such as proteases, amylases, and plant cell wall-degrading enzymes (Fig. 5, Fig. S7). Although both *A. oryzae* and *A. sojae* are used in soy sauce brewing, previous studies have reported differences in enzymatic activities related to soy sauce brewing (Ito and Matsuyama 2021). Differences in the characteristics of *A. oryzae* and *A. sojae* may have contributed to the slight variations in koji mold–derived proteins detected in the heat-induced sediment.

**Fig. 6:**
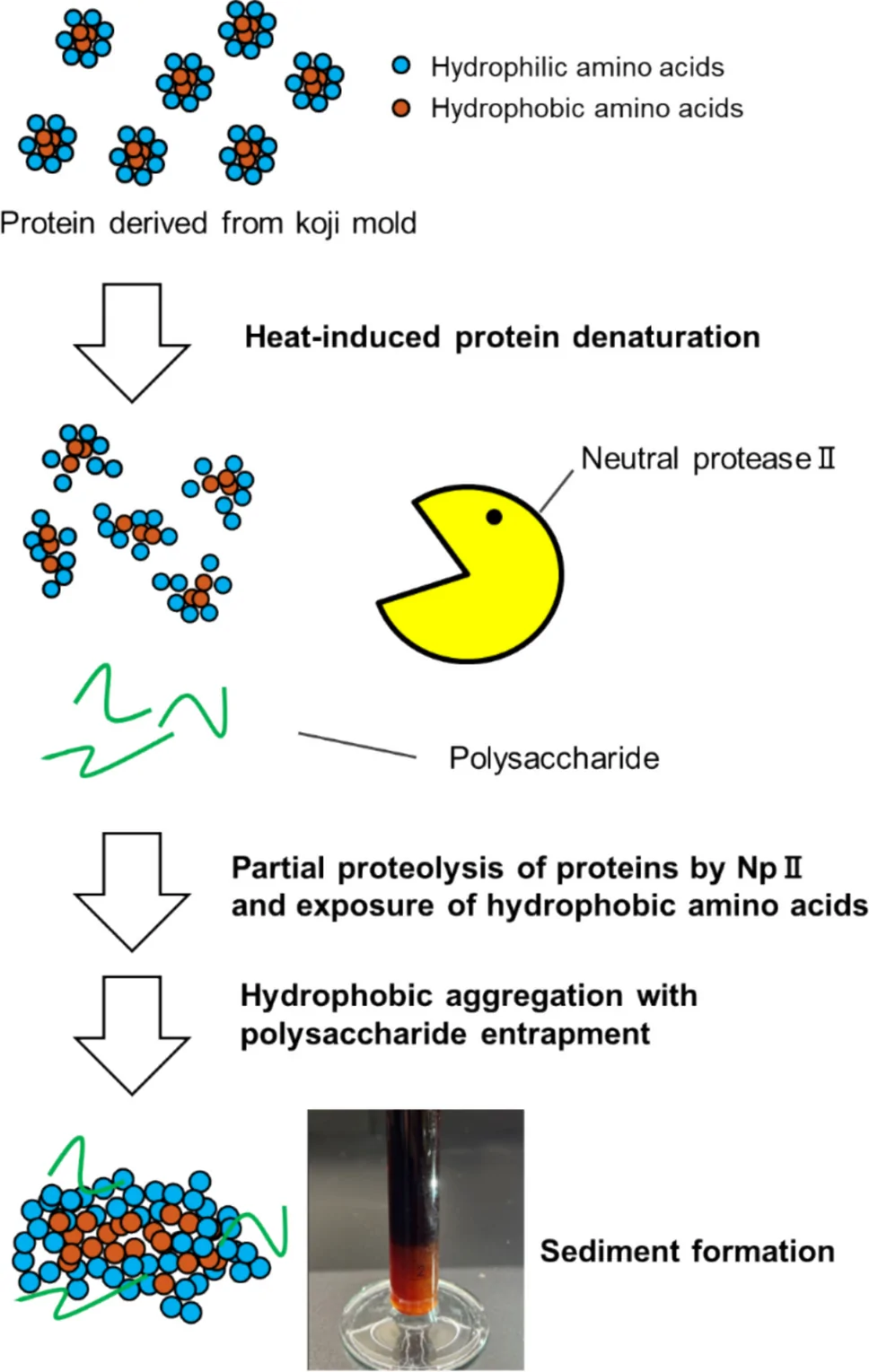
Proposed working model of heat-induced sediment formation in soy sauce. Model is based on indirect experimental evidence and is intended to illustrate a plausible mechanism

Based on these findings, the heat-induced sediment formation mechanism appears to be best explained by the model proposed by Hashimoto and Yokotsuka (1974a; b). During the first step of soy sauce brewing, koji molds produce large amounts of enzymes that contribute to soy sauce production. These enzymes are thought to undergo partial denaturation during the fermentation process due to factors such as pH reduction by lactic acid bacteria and alcohol production by yeast and are subsequently self-digested by proteases during fermentation. Proteins, such as enzymes, that escape this autolysis and remain stable until just before heat treatment are then denatured at 80 °C, thereby becoming susceptible to proteolytic degradation. While most proteases are inactivated by this heat treatment owing to their low thermostability, the thermostable NpII is not completely inactivated at this temperature and acts on the denatured koji mold–derived proteins that serve as the basis of the sediment. Taken together with the results shown in Fig. 3b, these findings indicate that proteolytic activity during heating is not essential for sediment formation and that NpII predominantly exerts its effect after heat-induced protein denaturation. The resulting peptides had exposed hydrophobic amino acid residues, which gradually aggregated while entrapping polysaccharides (Sonehara and Hayashi 1982), ultimately forming heat-induced sediments (Fig. 6). In addition to sediment formation, the impact of npII disruption on soy sauce composition is an important consideration from an industrial perspective. In the present study, soy sauce produced using Δ*npII* strains showed no significant differences in total nitrogen content or amino acid composition compared with the control strain. These results indicate that deletion of npII does not markedly affect overall protein degradation or amino acid release at the level detectable by bulk compositional analysis. Although sensory evaluation was not performed in this study, the absence of major changes in these fundamental compositional parameters suggests that the contribution of NpII to flavor-related components may be limited. Nevertheless, subtle effects on aroma, taste, or mouthfeel cannot be excluded, and future studies incorporating sensory analysis will be necessary to fully assess the impact of npII disruption on soy sauce quality.

Protein aggregation, which leads to haze, sediment formation, and gelation, is a critical issue that often compromises the commercial value and sensory quality of food products. For instance, wine haze is believed to result from the gradual denaturation and aggregation of grape proteins, such as thaumatin-like proteins and chitinases, during storage, a process influenced by factors, including sulfates, phenolic compounds, and polysaccharides, through mechanisms that remain incompletely understood (Cosme et al. 2020; Van Sluyter et al. 2015). Protein removal is considered an effective method for preventing haze formation during wine brewing. Among these methods, bentonite fining, in which proteins are adsorbed onto bentonite and removed as precipitates, has been widely used in commercial applications. However, bentonite fining can reduce aroma compounds and decrease yield, prompting interest in alternative approaches, such as the addition of protease preparations to degrade haze-forming proteins (Marangon et al. 2012; Theron et al. 2018; Van Sluyter et al. 2013). For example, the use of aspergillopepsin I combined with heat treatment effectively reduced wine haze. This method has been approved by the International Organization of Vine and Wine, although its use remains limited because the operating conditions must be adapted to the type of wine (Filipe-Ribeiro et al. 2022; Silva-Barbieri et al. 2022). In contrast to the mechanism observed in wine protein aggregation, this study demonstrated that during soy sauce brewing, proteases act as triggers for protein aggregation, promoting heat-induced sediment formation. Similarly, in ultra-high temperature (UHT)–treated milk, heat-resistant exogenous proteases of bacterial origin are likely to remain active after thermal processing, resulting in sediment formation or gelation during storage (Baglinière et al. 2017; Krishna et al. 2021; Qin et al. 2023). Recent experiments evaluating the reactivity of various proteases toward wine proteins under heat treatment have suggested that some proteases may even enhance haze formation compared to conditions without protease addition (Seidel et al. 2025). These findings indicate that proteases can function in food production not only to prevent protein aggregation but also to promote it. Future investigations into changes in protein aggregation behavior associated with variations in protease profiles may provide insights into the mechanisms underlying haze and sediment formation in foods other than soy sauce.

In this study, we demonstrated that the major component of heat-induced sediment in soy sauce is koji mold–derived proteins, and that sediment formation is promoted by the thermostable protease NpII produced by this fungus. Furthermore, soy sauce produced using the *npII*-disruptant strain showed no heat-induced sedimentation after sterilization, and its chemical properties, including total nitrogen and amino acid concentrations, were comparable to those of soy sauce prepared using the control strain. Using the *npII*-disruptant strain represents a practical strategy for soy sauce production by substantially reducing the need for processes to remove heat-induced sediments.

## Supplementary Information

Below is the link to the electronic supplementary material.

ESM 1 Supplementary Material 1 (PDF 822 KB)

ESM 2 Supplementary Material 2 (PDF 216 KB)

ESM 3 Supplementary Material 3 (XLSX 259 KB)

ESM 4 Supplementary Material 4 (XLSX 340 KB)

ESM 5 Supplementary Material 5 (XLSX 178 KB)

## Data Availability

The results of the proteomic analyses were accessed using jPOST (accession ID will be provided upon acceptance). Other data generated or analyzed during this study are included in this published article and its supplementary information files.
