# Supplementary material for: Role of Aspergillus neutral protease II in heat-induced soy sauce sediment formation
Source: Appl Microbiol Biotechnol. 2026 Jul 3;110:285. doi: 10.1007/s00253-026-13923-w (PMC13601390; doi:10.1007/s00253-026-13923-w)
Supplement: Supplementary file 1 [file 253_2026_13923_MOESM1_ESM.pdf]

Supplementary Information

Title:

Role of *Aspergillus* Neutral Protease II in Heat-Induced Soy Sauce Sediment Formation

Authors:

Takayuki Igarashi, Hideo Ikeuchi, Naho Shinohara, Yuichiro Hashiguchi, Yasuko Araki, and Kotaro Ito*

Research & Development Division, Kikkoman Corporation, 338 Noda, Noda, Chiba, 278-0037, Japan

*Email address: koito@mail.kikkoman.co.jp

Journal:
Applied Microbiology and Biotechnology

- **This Supplementary Information includes the following materials:**
- **Supplementary Table S1. List of the strains used in this study**
- **Supplementary Table S2. List of oligonucleotides used in this study**
- **Supplementary Table S3. Complete list of proteins detected in raw soy sauce and heat-induced sediment produced with *Aspergillus oryzae* RIB40, identified by DIA-based proteomic analysis. (Excel file)**
- **Supplementary Table S4. Complete list of proteins detected in raw soy sauce and heat-induced sediment produced with *Aspergillus oryzae* RIB326, identified by DIA-based proteomic analysis. (Excel file)**
- **Supplementary Table S5. Complete list of proteins detected in raw soy sauce and heat-induced sediment produced with *Aspergillus sojae* NBRC4239, identified by DIA-based proteomic analysis. (Excel file)**
- **Supplementary Figure S1. Nucleotide and amino acid sequences of the *npII* gene derived from *Aspergillus sojae***
- **Supplementary Figure S2. Enzyme activities of koji produced using the control strains and the Δ*npII* strains**
- **Supplementary Figure S3. Evaluation of heat-induced soy sauce sediment produced by strains complemented with either wild-type or mutated *npII* genes**
- **Supplementary Figure S4. Chemical composition of soy sauce produced using the control strains and the Δ*npII* strains**
- **Supplementary Figure S5. Amino acid concentrations in soy sauce produced using control strains and Δ*npII* strains**
- **Supplementary Figure S6. SDS-PAGE analysis of the NpII enzyme solution**
- **Supplementary Figure S7. The top 30 proteins detected in raw soy sauce and heat-induced sediment (*Aspergillus oryzae* RIB40)**
- **Supplementary Figure S8. The top 30 proteins detected in raw soy sauce and heat-induced sediment (*Aspergillus oryzae* RIB326)**
- **Supplementary Figure S9. The top 30 proteins detected in raw soy sauce and heat-induced sediment (*Aspergillus sojae* NBRC4239)**

Table S1. Strains used in this study

Name	Genotype	Refernces
A. sojae **NBRC4239**		
KP-del	Δ*pyrG* Δ*ku70*	Araki et al., 2021
P6-1	*niaD*- Δ*pyrG*::*niaD*	Araki et al., 2021
Δ*npII*	Δ*pyrG* Δ*ku70* Δ*npII*::*pyrG*	This study
K-del	Δ*ku70*	This study
As-npII_OE	*niaD*- Δ*pyrG*::*niaD* pUC19-pG56-npII	This study
As-npII-WT_CP	Δ*pyrG* Δ*ku70* Δ*npII*::*pyrG* Δ*Asgdh*::*ptrA*-P*npII*-*npII*(WT)-T*npII*	This study
As-npII-mut1_CP	Δ*pyrG* Δ*ku70* Δ*npII*::*pyrG* Δ*Asgdh*::*ptrA*-P*npII*-*npII*(mutation1)-T*npII*	This study
As-npII-mut2_CP	Δ*pyrG* Δ*ku70* Δ*npII*::*pyrG* Δ*Asgdh*::*ptrA*-P*npII*-*npII*(mutation2)-T*npII*	This study
A. oryzae **RIB40**		
KP-del (AoXku70::ptrA)	Δ*pyrG* Δ*ku70*::*ptrA*	Takahashi et al., 2006
Δ*npII*	Δ*pyrG* Δ*ku70*::*ptrA* Δ*npII*::*pyrG*	This study
K-del	Δ*ku70*::*ptrA*	This study
A. oryzae **RIB326**		
KP-del	Δ*pyrG* Δ*ku70*::*ptrA*	This study
Δ*npII*	Δ*pyrG* Δ*ku70*::*ptrA* Δ*npII*::*pyrG*	This study
K-del	Δ*ku70*::*ptrA*	This study

Table S2. Oligonucleotides used in this study

Name	Sequence (5' to 3')	Gene
**Construction of *A. sojae* Δ*npII***		
pUC19-npII_5arm-Fw_As	CGGTACCCGGGGATCCCACTAGATGTATAGGTGGTGACG	5'-upstream
npII_5arm-Rv_As	TTAGTCAAGGGCTGTGTTGGGCTCGGAATAAG	
npII_5arm-pyrG-Fw_As	ACAGCCCTTGACTAATTGGGCTTATTGCTATGTCCCTG	*pyrG* marker
pyrG-npII_3arm-Rv_As	CCAGATCAAAGCACTCCCGCACCTCAGAAGAAAAGGAT	
npII_3arm-Fw_As	GAGTGCTTTGATCTGGTGAATCGCCAAAGG	3'-downstream
npII_3arm-pUC19-Rv_As	CGACTCTAGAGGATCGATAACTTCACTAAGCTCATCCCAAG	
**Construction of *A. oryzae* Δ*npII***		
pUC19-npll-5arm-Fw	CGGTACCCGGGGATCCGATTCCCCAAGAAGCAATATGAC	5'-upstream
npll-5arm-Rv	CTGCCCTCCGTCTCATACTCTACCTTG	
npll-5arm-pyrG-Fw	TGAGACGGAGGGCAGTTGGGCTTATTGCTATGTCCCTG	*pyrG* marker
pyrG-npll-3arm-Rv	AGACATCTGGATTGACCGCACCTCAGAAGAAAAGGAT	
npll-3arm-Fw	TCAATCCAGATGTCTGGTGTGCTTTGATC	3'-downstream
npll-3arm-PUC19-Rv	CGACTCTAGAGGATCCATTTTACAAACTTGGGC	
**Construction of the *npII*-overexpressing strain in *A. sojae***		
HCNp-Fw	TCGCACCACCTTCAAAATGCGTGTCACTGCTCTCTCCACC	*npII*
HCNp_Rv	ATGTACTCCTGGTACTTAGCACTTGAGCTCGATAGCTATT	
Talp-F1	GTACCAGGAGTACATTGGAGAGTTC	vector
Ptef-R1	TTTGAAGGTGGTGCGAACTTTGTAG	
**Construction of the complemented strain in *A. sojae* Δ*npII***		
Tnp-GDH-3arm-1F	AAGTTGTCGATGTTCAGAGGTGTGCTAGAT	vector
GDH-5arm-PT-1R	ATTGCCCCTTGGTGATAGATAGATGCGAGG	
GDH-5arm-PT-2F	TCACCAAGGGGCAATTGATTACGGGATCCC	*ptrA*
PT-Pnp-2R	GCTGTAGCATGGGGTGACGATGAGCCGCTC	
PT-Pnp-3F	ACCCCATGCTACAGCTGCGCCCATCATTAT	P*npII*-*npII*-T*npII*
Tnp-GDH-3arm-3R	GAACATCGACAACTTCGAAGGATCTACTCT	
npII-mut1-Fw	GTCAAGCTGACCtAGATTGACAACACTCGT	For the introduction of the *npII* mutation
npII-mut1-Rv	GACAACCGCCTTCACACGAGTGTTGTCAAT	
npII-mut2-Fw	GTCTACCGCGGCtGTACGTTTCCCTTTAAC	
npII-mut2-Rv	TTCAATGACTAGTCTGTTAAAGGGAAACGT	
GDH-5arm-Fw	CGAGACCTGTGAGCCTATCAAGGATC	(For transformation)
GDH-3arm-Rv	GGTTCGTTCAAGGCAGCCAAATTCG	

The sequence necessary for In-Fusion reaction are underlined.

```
            10        20        30        40        50        60
    ATGCGTGTCACTGCTCTCTCCACCGCACTCTTCGCGTTGGCTTCCACCGCTGTGTCGGCG
1   M  R  V  T  A  L  S  T  A  L  F  A  L  A  S  T  A  V  S  A      20
                                                 T(mutation1)
            70        80        90       100      ↑110       120
    CCAACCGCTGGGAACTCGTCTCCTGGTCTTGAGGTCAAGCTGACCCAGATTGACAACACT
21  P  T  A  G  N  S  S  P  G  L  E  V  K  L  T  Q  I  D  N  T      40

           130       140       150       160       170       180
    CGTGTGAAGGCGGTTGTCAAGAACACCGGCAGTGAGGAAGTGTCCTTCGTCCACCTGAAC
41  R  V  K  A  V  V  K  N  T  G  S  E  E  V  S  F  V  H  L  N      60
                                                    T(mutation2)
           190       200       210       220       2↑0       240
    TTCTTCAAGGACGCTGGCCCTGTCAAGAAGGTTTCCGTCTACCGCGGCCGTACGTTTCCC
61  F  F  K  D  A  G  P  V  K  K  V  S  V  Y  R  G  Q               77

           250       260       270       280       290       300
    TTTAACAGACTAGTCATTGAATATCAATGGCGTTTATTAACATTCCCGCAGAGGATGAGG
78                                                      D  E  V      80

           310       320       330       340       350       360
    TCCAGTTCGAGGGTATCAAGCGCCGCTTGAGGAGCAGTGGCATCACTAAGGAGGCTGTCA
81   Q  F  E  G  I  K  R  R  L  R  S  S  G  I  T  K  E  A  V  T     100

           370       380       390       400       410       420
    CTTCTCTTGGTGCCGGTGAGACCCTGGAGGATGAGTTCGATATCGCCTCCACCAGCGACC
101  S  L  G  A  G  E  T  L  E  D  E  F  D  I  A  S  T  S  D  L    120

           430       440       450       460       470       480
    TGGCAAGCGGCGGTCCCGTTTCCATCCGCAGCCACGGATTCGTCCCCATTGTCGTGGACG
121  A  S  G  G  P  V  S  I  R  S  H  G  F  V  P  I  V  V  D  G    140

           490       500       510       520       530       540
    GCAAGATCACCGGCTATATCCCTTACAAGTCCAACGATCTGACAGTCAATGTTGACGGCG
141  K  I  T  G  Y  I  P  Y  K  S  N  D  L  T  V  N  V  D  G  G    160

           550       560       570       580       590       600
    GCAAGGCCGCCAAGGTTACCAAGGCGCTGTCGCAGCTCACTCGCCGCACCGAGGTCACTG
161  K  A  A  K  V  T  K  A  L  S  Q  L  T  R  R  T  E  V  T  D    180

           610       620       630       640       650       660
    ACTGCAAGGGCGACGCCGAGTCCTCGTTGACCACTGCCCTTTCCAACGCTGCCAAGCTGG
181  C  K  G  D  A  E  S  S  L  T  T  A  L  S  N  A  A  K  L  A    200

           670       680       690       700       710       720
    CCAACCAGGCTGCCGAAGCCGCCGAATCCGGCGACGAGTCTAAGTTCGAGGAGTACTTCA
201  N  Q  A  A  E  A  A  E  S  G  D  E  S  K  F  E  E  Y  F  K    220

           730       740       750       760       770       780
    AGACCACCGACAAGCAGACCCGCACGACCGTTGCTGAGCGTCTGCGCGCTGTCGCCAAGG
221  T  T  D  K  Q  T  R  T  T  V  A  E  R  L  R  A  V  A  K  E    240

           790       800       810       820       830       840
    AAGCCGGCTCTACCTCCGGTGGCTCTACCACGTACCACTGCAACGACCCCTACGGCTACT
241  A  G  S  T  S  G  G  S  T  T  Y  H  C  N  D  P  Y  G  Y  C    260

           850       860       870       880       890       900
    GTGAGCCGAACGTTCTGGCCTACACCCTGCCCTCGAAGAACGAGATCGCCAACTGCGACA
261  E  P  N  V  L  A  Y  T  L  P  S  K  N  E  I  A  N  C  D  I    280

           910       920       930       940       950       960
    TCTACTACTCTGAGCTTCCTCCCCTGGCTCAGAAGTGCCACGCCCAGGACCAGGCCACCA
281  Y  Y  S  E  L  P  P  L  A  Q  K  C  H  A  Q  D  Q  A  T  T    300

           970       980       990      1000      1010      1020
    CTACTCTTCACGAGTTCACTCACGCCCCTGGCGTCTACCAGCCTGGCACTGAGGACTTGG
301  T  L  H  E  F  T  H  A  P  G  V  Y  Q  P  G  T  E  D  L  G    320

          1030      1040      1050      1060      1070      1080
    GCTACGGCTATGATGCCGCTACTCAGCTGAGCGCCCAGGATGCGTTGAACAACGCTGATT
321  Y  G  Y  D  A  A  T  Q  L  S  A  Q  D  A  L  N  N  A  D  S    340

          1090      1100      1110      1120      1130      1140
    CCTATGCCTTGTACGCCAATGGTAAGCATGTCCCGATAATATCTATTTGGTGCAAACAGT
341  Y  A  L  Y  A  N  A                                            347

          1150      1160      1170
    TGCTGATTGATAATAGCTATCGAGCTCAAGTGCTAA
348                  I  E  L  K  C  *     353
```

Fig. S1

Nucleotide and amino acid sequences of the *npII* gene derived from *Aspergillus sojae*. Mutations introduced in the Δ*npII* expression cassettes of As-npII-mut1_CP and As-npII-mut2_CP are indicated as mutations 1 and 2, respectively. Introns are highlighted in yellow.

Fig. S1 Takayuki Igarashi *et al.*

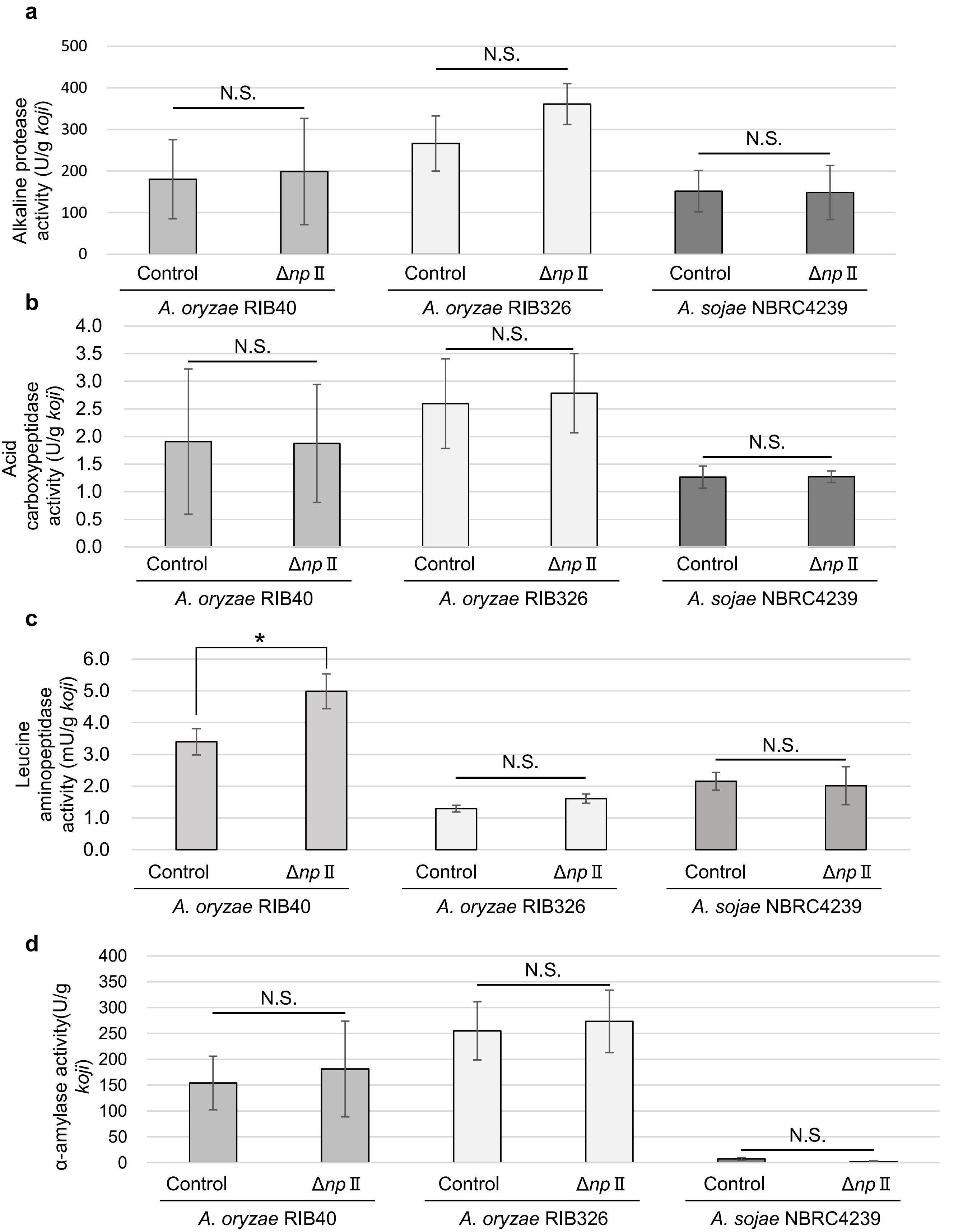

Fig. S2
Enzyme activities of koji produced using the control strains and the Δ*npII* strains.
a Alkaline protease activity, **b** Acid carboxypeptidase activity, **c** Leucine aminopeptidase activity, **d** α-amylase activity. Enzyme activities were measured using standard assay kits after koji cultivation under identical conditions. Data are presented as the means of three independent experiments. Error bars indicate standard deviation. Statistical significance was evaluated by Welch's t-test. Asterisks indicate statistically significant differences (*$p < 0.05$), and N.S. indicates no significant difference.

Fig. S2 Takayuki Igarashi *et al.*

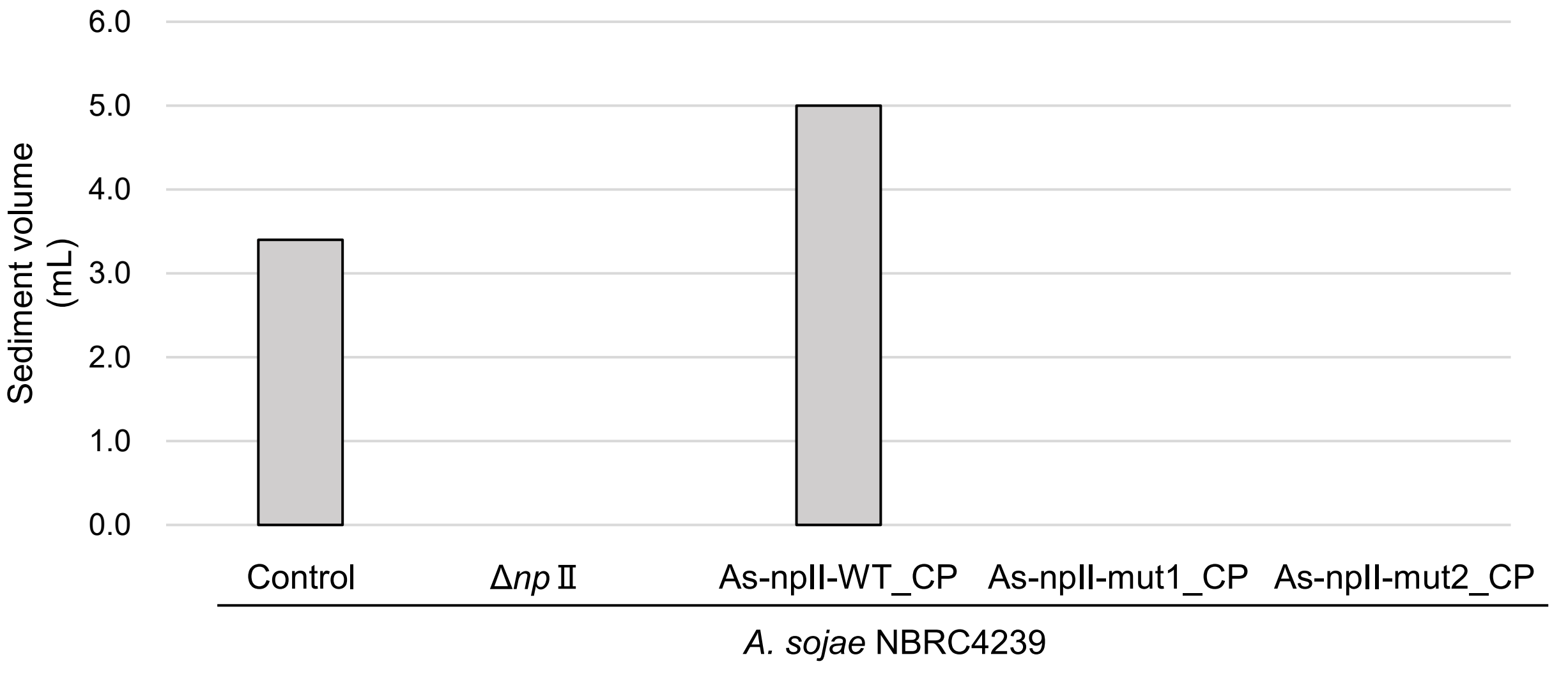

Fig. S3
Evaluation of heat-induced soy sauce sediment produced by strains complemented with either wild-type or mutated *npII* genes (As-npII-WT_CP, As-npII-mut1_CP, and As-npII-mut2_CP). Raw soy sauce was heat-treated under the same conditions as described in the main text, and sediment formation was evaluated after incubation. Data shown are from a single experiment.

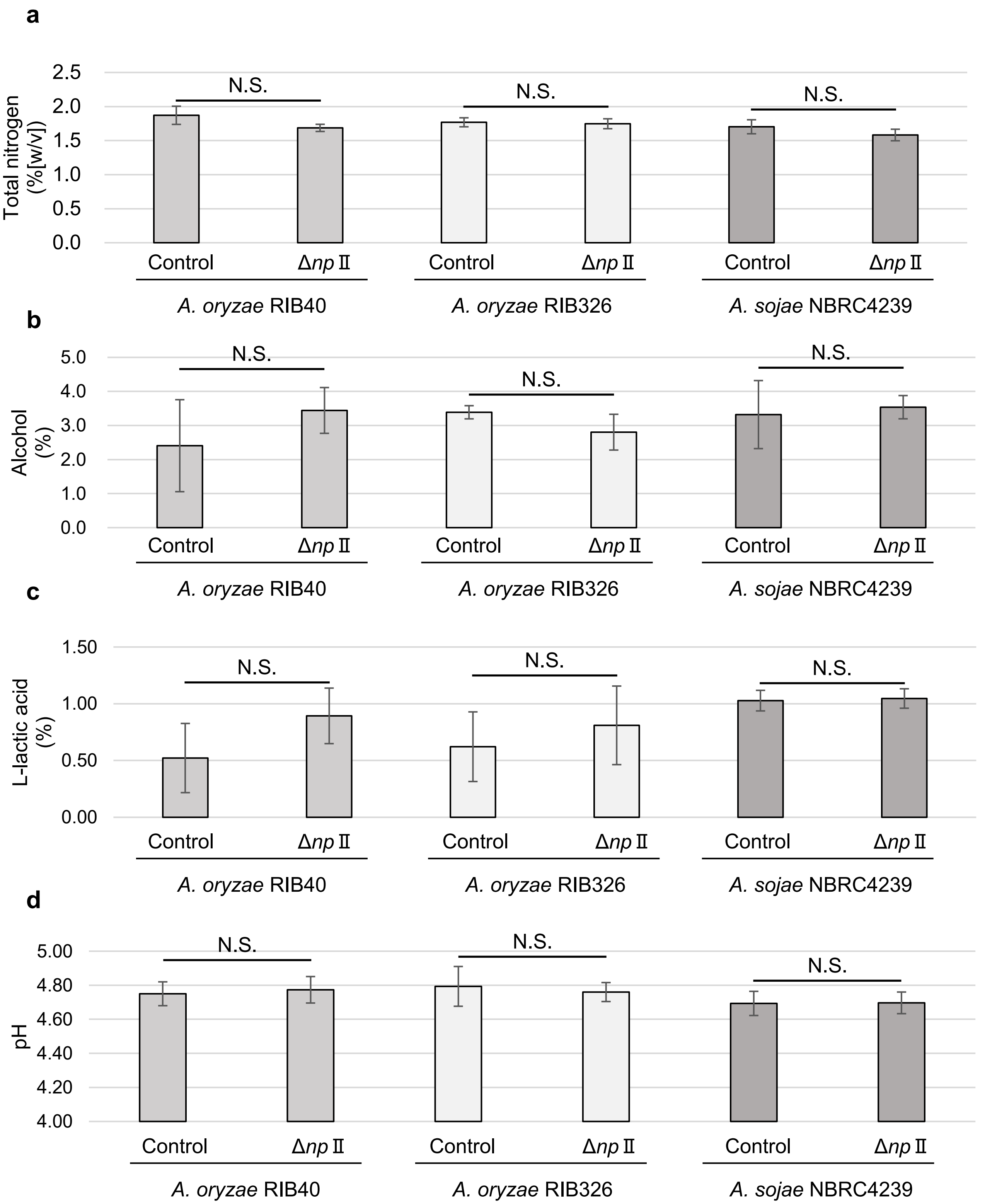

Fig. S4

Chemical composition of soy sauce produced using the control strains and the Δ*npII* strains. **a** Total nitrogen (TN) content, **b** alcohol content, **c** l-lactic acid content, **d** pH. Data are presented as the means of three independent experiments. Error bars indicate standard deviation. Statistical significance was evaluated by Welch's t-test. N.S. indicates no significant difference between Control and Δ*npII* strains.

Fig. S4 Takayuki Igarashi *et al.*

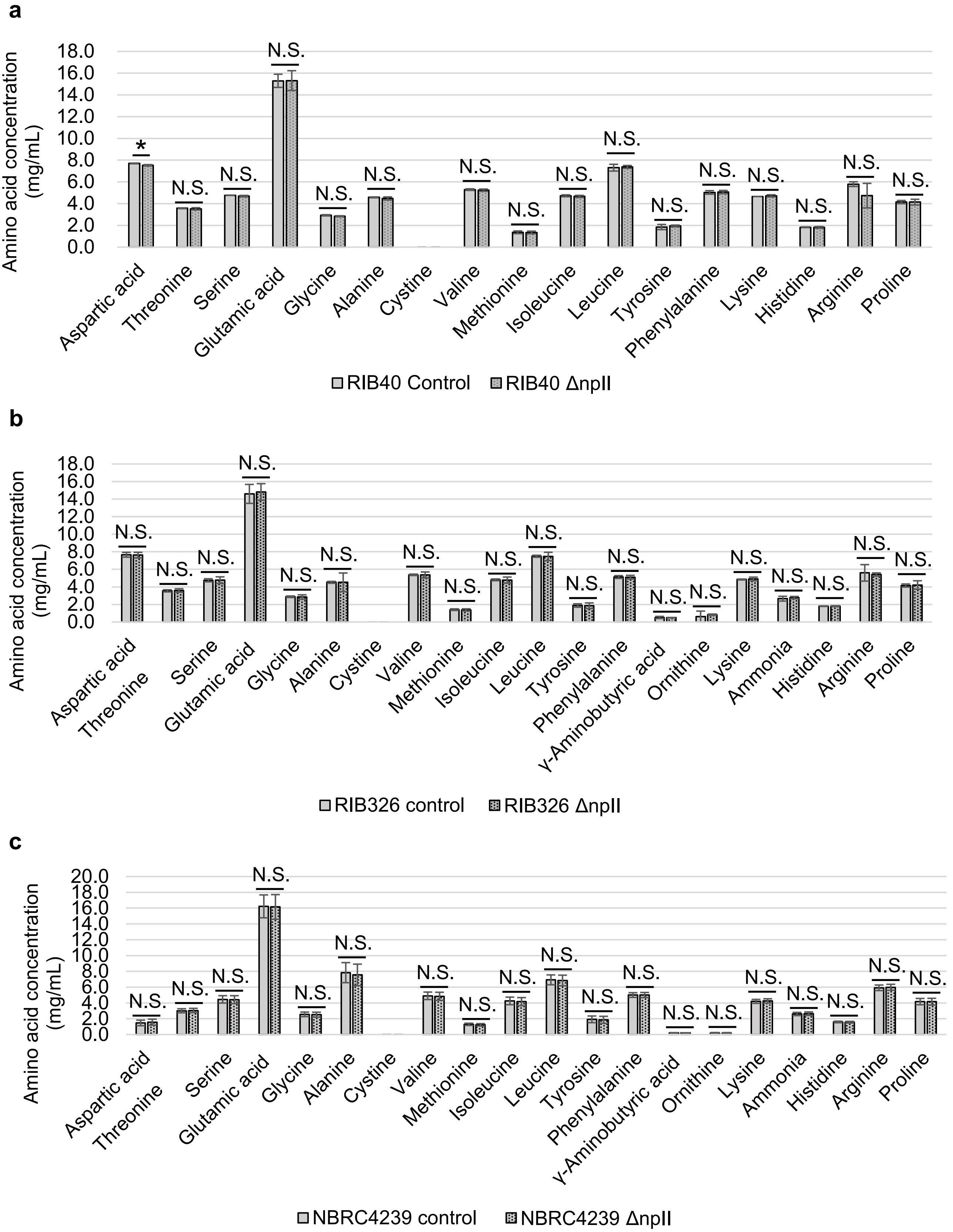

Fig. S5

Amino acid concentrations in soy sauce produced using control strains and Δ*npII* strains.

Amino acid concentrations were normalized to a TN content of 1.8% (w/v) in the soy sauce produced. **a** *A. oryzae* RIB40, **b** *A. oryzae* RIB326, **c** *A. sojae* NBRC4239 strains. Data are presented as means of three independent experiments. Error bars indicate standard deviation. Statistical significance was evaluated by Welch's t-test. Asterisks indicate statistically significant differences (*P < 0.05), and N.S. indicates no significant difference.

Fig. S5 Takayuki Igarashi *et al.*

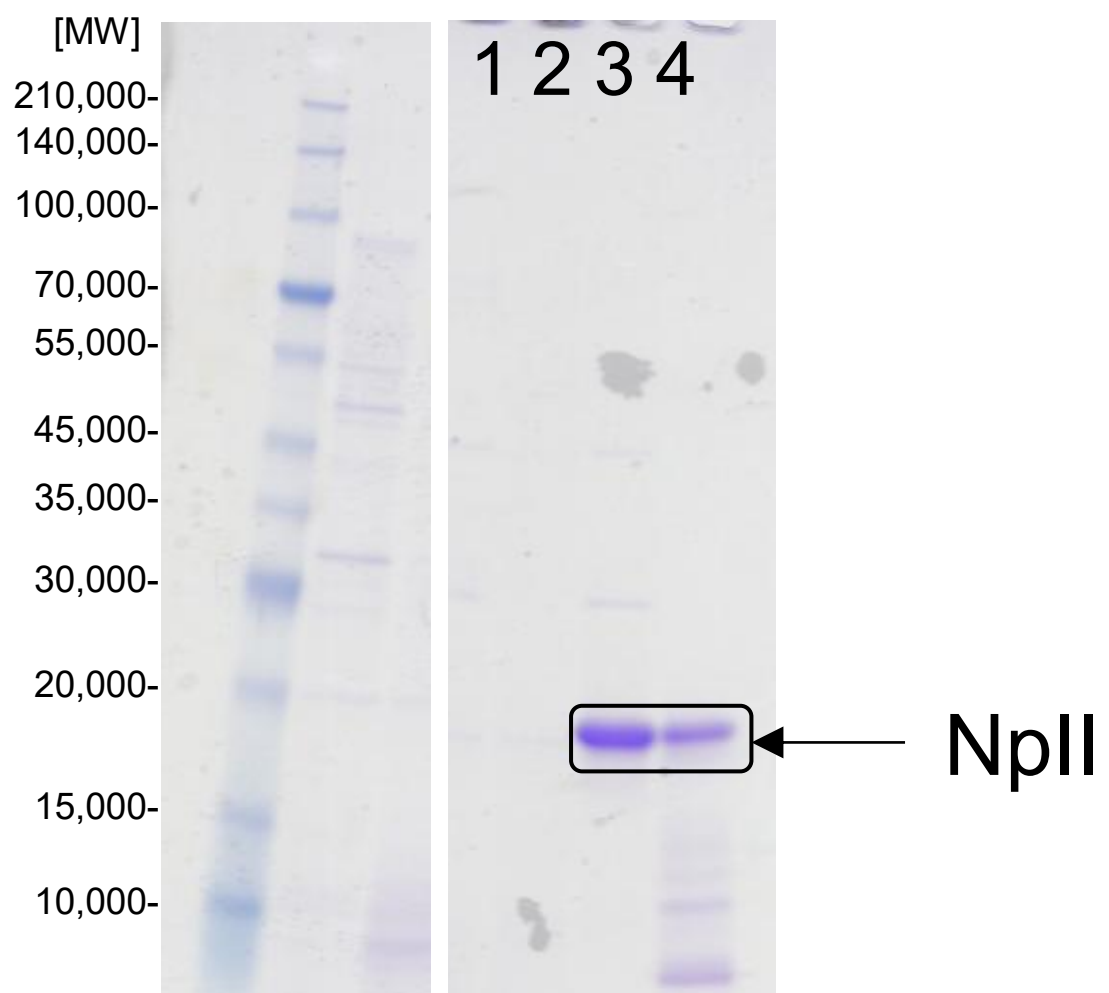

Fig. S6
SDS-PAGE analysis of the NpII enzyme solution. Proteins were separated by SDS-PAGE and visualized by Coomassie Brilliant Blue staining. As-npII-OE was cultivated in the Czapek-Dox liquid minimal medium for 7 days at 30 °C, and the culture broth was concentrated using a 10K Amicon ultrafiltration membrane. Heat treatment was performed at 80 °C for 30 min.
Lane 1: P6-1, Lane 2 : P6-1 (heated), Lane 3 : As-npII_OE, Lane 4 : As-npII_OE (heated)

Fig. S6 Takayuki Igarashi *et al.*

No.	Protein name/Gene Symbol/Speicies	
	raw soy sauce	sediment
1	glucan 1,4-alpha-glucosidase/AO090003000321/A. oryzae	glucan 1,4-alpha-glucosidase/AO090003000321/A. oryzae
2	Aspergillopepsin-1/pepA/A. oryzae	Aspergillopepsin-1/pepA/A. oryzae
3	Catalase B/catB/A. oryzae	Dipeptidyl-peptidase 5/AO090011000795-A/A. oryzae
4	Dipeptidyl-peptidase 5/AO090011000795-A/A. oryzae	Catalase B/catB/A. oryzae
5	Leucine aminopeptidase A/lapA/A. oryzae	Carboxypeptidase/AO090012000706/A. oryzae
6	Carboxypeptidase/AO090012000706/A. oryzae	tripeptidyl-peptidase II/AO090011000235/A. oryzae
7	Glucoamylase/glaA/A. oryzae	Leucine aminopeptidase A/lapA/A. oryzae
8	Glycinin G1/GY1/Glycine max	Glucoamylase/glaA/A. oryzae
9	Extracellular metalloproteinase Npl/Npl/A. oryzae	Extracellular metalloproteinase Npl/Npl/A. oryzae
10	tripeptidyl-peptidase II/AO090011000235/A. oryzae	Alpha-glucosidase/AO090038000471/A. oryzae
11	alkaline phosphatase/AO090012000725/A. oryzae	alkaline phosphatase/AO090012000725/A. oryzae
12	Purine nucleoside permease/AO090701000344/A. oryzae	Carboxypeptidase/AO090010000534/A. oryzae
13	Alpha-amylase A type-3/amy3/A. oryzae	Probable beta-glucosidase A/bglA/A. oryzae
14	ThiJ/Pfpl domain-containing protein/AO090012000695/A. oryzae	FAD-binding PCMH-type domain-containing protein/AO090001000617/A. oryzae
15	FAD/NAD(P)-binding domain-containing protein/AO090102000564/A. oryzae	Purine nucleoside permease/AO090701000344/A. oryzae
16	Neutral protease 2/AO090010000493/A. oryzae	tripeptidyl-peptidase II/AO090166000084/A. oryzae
17	Alpha-glucosidase/AO090038000471/A. oryzae	Alkaline protease 1/alp1/A. oryzae
18	Probable beta-glucosidase A/bglA/A. oryzae	Carboxypeptidase/AO090701000220/A. oryzae
19	Elongation factor Tu/tuf/Tetragenococcus halophilus subsp. halophilus	Beta-hexosaminidase/AO090005000639/A. oryzae
20	Enolase/eno/Tetragenococcus halophilus subsp. halophilus	Carboxypeptidase M14B/AO090120000008/A. oryzae
21	Glycinin G4/GY4/Glycine max	beta-glucosidase/AO090701000274/A. oryzae
22	probable leucine aminopeptidase 2/lap2/A. oryzae	probable leucine aminopeptidase 2/lap2/A. oryzae
23	Aldose 1-epimerase/AO090012000809/A. oryzae	Probable beta-galactosidase B/lacB/A. oryzae
24	Hydrolase/AO090023000053/A. oryzae	Peptidase S28/AO090003000876/A. oryzae
25	1,3-beta-glucanosyltransferase/AO090001000635/A. oryzae	FAD-binding PCMH-type domain-containing protein/AO090005000347/A. oryzae
26	Carboxypeptidase M14B/AO090120000008/A. oryzae	FAD/NAD(P)-binding domain-containing protein/AO090102000564/A. oryzae
27	beta-glucosidase/AO090701000274/A. oryzae	Hydrolase/AO090023000053/A. oryzae
28	tripeptidyl-peptidase II/AO090166000084/A. oryzae	Beta-galactosidase A/lacA/A. oryzae
29	Beta-hexosaminidase/AO090005000639/A. oryzae	1,3-beta-glucanosyltransferase/AO090001000635/A. oryzae
30	Beta-galactosidase A/lacA/A. oryzae	Acid phosphatase/AO090011000300/A. oryzae

Fig. S7
Top 30 proteins detected in raw soy sauce and heat-induced sediment produced using *Aspergillus oryzae* RIB40, as identified by data-independent acquisition (DIA)–based proteomic analysis.
Proteins were ranked based on normalized values obtained by dividing the sum of the unique peptide intensities by the molecular weight of each protein. Raw soy sauce and heat-induced sediment are shown in separate columns. Proteins identified as identical by DIA analysis are indicated in the same color.

Fig. S7 Takayuki Igarashi *et al.*

No.	Protein name/Gene Symbol/Speicies	
	raw soy sauce	sediment
1	Aspergillopepsin-1/pepA/A. oryzae	glucan 1,4-alpha-glucosidase/AO090003000321/A. oryzae
2	glucan 1,4-alpha-glucosidase/AO090003000321/A. oryzae	Dipeptidyl-peptidase 5/AO090011000795-A/A. oryzae
3	Dipeptidyl-peptidase 5/AO090011000795-A/A. oryzae	Aspergillopepsin-1/pepA/A. oryzae
4	Leucine aminopeptidase A/lapA/A. oryzae	Carboxypeptidase/AO090012000706/A. oryzae
5	Catalase B/catB/A. oryzae	Catalase B/catB/A. oryzae
6	Carboxypeptidase/AO090012000706/A. oryzae	Leucine aminopeptidase A/lapA/A. oryzae
7	Extracellular metalloproteinase Npl/Npl/A. oryzae	tripeptidyl-peptidase II/AO090011000235/A. oryzae
8	Purine nucleoside permease/AO090701000344/A. oryzae	Extracellular metalloproteinase Npl/Npl/A. oryzae
9	tripeptidyl-peptidase II/AO090011000235/A. oryzae	Purine nucleoside permease/AO090701000344/A. oryzae
10	Aldose 1-epimerase/AO090012000809/A. oryzae	tripeptidyl-peptidase II/AO090166000084/A. oryzae
11	alkaline phosphatase/AO090012000725/A. oryzae	Carboxypeptidase/AO090701000220/A. oryzae
12	Glycinin G1/GY1/G. max	Carboxypeptidase M14B/AO090120000008/A. oryzae
13	probable leucine aminopeptidase 2/lap2/A. oryzae	alkaline phosphatase/AO090012000725/A. oryzae
14	tripeptidyl-peptidase II/AO090166000084/A. oryzae	FAD-binding PCMH-type domain-containing protein/AO090001000617/A. oryzae
15	Carboxypeptidase M14B/AO090120000008/A. oryzae	Probable beta-glucosidase A/bglA/A. oryzae
16	Small ribosomal subunit protein bS21/rpsU/T. halophilus	Glucoamylase/glaA/A. oryzae
17	Carboxypeptidase/AO090701000220/A. oryzae	Carboxypeptidase/AO090010000534/A. oryzae
18	Glucoamylase/glaA/A. oryzae	BNR/Asp-box repeat domain protein/AO090003001017/A. oryzae
19	Probable beta-glucosidase A/bglA/A. oryzae	Alpha-glucosidase/AO090038000471/A. oryzae
20	Ribonuclease T2/rntB/A. oryzae	probable leucine aminopeptidase 2/lap2/A. oryzae
21	Phosphoesterase/AO090012000083/A. oryzae	Beta-hexosaminidase/AO090005000639/A. oryzae
22	Neutral protease 2/AO090010000493/A. oryzae	FAD-binding PCMH-type domain-containing protein/AO090005000347/A. oryzae
23	Alpha-glucosidase/AO090038000471/A. oryzae	Hydrolase/AO090023000053/A. oryzae
24	ThiJ/PfpI domain-containing protein/AO090012000695/A. oryzae	Probable beta-galactosidase B/lacB/A. oryzae
25	Hydrolase/AO090023000053/A. oryzae	Phosphoesterase/AO090012000083/A. oryzae
26	ATP synthase subunit c/atpE/T. halophilus	Beta-galactosidase A/lacA/A. oryzae
27	Glyceraldehyde-3-phosphate dehydrogenase/gap/T. halophilus	Phytase/AO090010000202/A. oryzae
28	Beta-hexosaminidase/AO090005000639/A. oryzae	Carboxypeptidase/AO090103000026/A. oryzae
29	Elongation factor Tu/tuf/T. halophilus	Nuclease S1/nucS/A. oryzae
30	Phytase/AO090010000202/A. oryzae	beta-glucosidase/AO090701000274/A. oryzae

Fig. S8

Top 30 proteins detected in raw soy sauce and heat-induced sediment produced using *Aspergillus oryzae* RIB326, as identified by data-independent acquisition (DIA)–based proteomic analysis.

Proteins were ranked based on normalized values obtained by dividing the sum of unique peptide intensities by the molecular weight of each protein. Raw soy sauce and heat-induced sediment are shown in separate columns. Proteins identified as identical by DIA analysis are indicated in the same color.

No.	Protein name/Gene Symbol/Speicies	
	raw soy sauce	sediment
1	Dipeptidyl-peptidase 5/AO090011000795-A/A. sojae	Dipeptidyl-peptidase 5/AO090011000795-A/A. sojae
2	Aldose 1-epimerase/AO090012000809/A. sojae	Probable beta-glucosidase A/bglA/A. sojae
3	Catalase B/catB/A. sojae	Catalase B/catB/A. sojae
4	Probable beta-glucosidase A/bglA/A. sojae	Alpha-glucosidase/AO090038000471/A. sojae
5	Alpha-glucosidase/AO090038000471/A. sojae	probable leucine aminopeptidase 2/lap2/A. sojae
6	Aspergillopepsin-1/pepA/A. sojae	BNR/Asp-box repeat domain protein/AO090003001017/A. sojae
7	Large ribosomal subunit protein uL23/rplW/T. halophilus	glucan 1,4-alpha-glucosidase/AO090003000321/A. sojae
8	glucan 1,4-alpha-glucosidase/AO090003000321/A. sojae	Aspergillopepsin-1/pepA/A. sojae
9	Purine nucleoside permease/AO090701000344/A. sojae	tripeptidyl-peptidase II/AO090011000235/A. sojae
10	Glycinin G1/GY1/G. max	Probable beta-galactosidase B/lacB/A. sojae
11	Leucine aminopeptidase A/lapA/A. sojae	Carboxypeptidase/AO090010000534/A. sojae
12	probable leucine aminopeptidase 2/lap2/A. sojae	Leucine aminopeptidase A/lapA/A. sojae
13	Probable beta-galactosidase B/lacB/A. sojae	non-reducing end alpha-L-arabinofuranosidase/AO090012000298/A. sojae
14	BNR/Asp-box repeat domain protein/AO090003001017/A. sojae	beta-glucosidase/AO090701000274/A. sojae
15	Probable mannan endo-1,4-beta-mannosidase A/manA/A. sojae	Purine nucleoside permease/AO090701000344/A. sojae
16	DNA-binding protein HU/hup/T. halophilus	Probable mannan endo-1,4-beta-mannosidase A/manA/A. sojae
17	Elongation factor Tu/tuf/T. halophilus	Extracellular metalloproteinase NpI/NpI/A. sojae
18	tripeptidyl-peptidase II/AO090011000235/A. sojae	Probable alpha-galactosidase D/aglD/A. sojae
19	non-reducing end alpha-L-arabinofuranosidase/AO090012000298/A. sojae	CBM6 domain-containing protein/AO090010000562/A. sojae
20	Neutral protease 2/AO090010000493/A. sojae	Beta-glucuronidase C-terminal domain-containing protein/AO090012000045/A. sojae
21	Uncharacterized protein/TEHN7118_0599/T. halophilus	Endopolygalacturonase I/pgaI/A. sojae
22	beta-glucosidase/AO090701000274/A. sojae	Probable alpha-galactosidase B/aglB/A. sojae
23	Large ribosomal subunit protein bL12/rplL/T. halophilus	Beta-hexosaminidase/AO090005000639/A. sojae
24	CBM6 domain-containing protein/AO090010000562/A. sojae	Peptidase S28/AO090003000876/A. sojae
25	Triosephosphate isomerase/tpiA/T. halophilus	Beta-galactosidase A/lacA/A. sojae
26	Extracellular metalloproteinase NpI/NpI/A. sojae	Aldose 1-epimerase/AO090012000809/A. sojae
27	Enolase/eno/T. halophilus	Nuclease S1/nucS/A. sojae
28	Carboxypeptidase/AO090010000534/A. sojae	Carboxypeptidase/AO090012000706/A. sojae
29	ThiJ/PfpI domain-containing protein/AO090012000695/A. sojae	beta-galactosidase/AO090001000259 /A. sojae
30	Endopolygalacturonase I/pgaI/A. sojae	SGNH hydrolase-type esterase domain-containing protein/AO090102000092/A. sojae

Fig. S9
Top 30 proteins detected in raw soy sauce and heat-induced sediment produced using *Aspergillus sojae* NBRC4239, as identified by data-independent acquisition (DIA)–based proteomic analysis. Proteins were ranked based on normalized values obtained by dividing the sum of unique peptide intensities by the molecular weight of each protein. Gene symbols of *A. sojae* were annotated based on the closest homologs in *A. oryzae*. Proteins identified as identical by DIA analysis are indicated in the same color.

Fig. S9 Takayuki Igarashi *et al.*
