# Supplementary material for: Role of Aspergillus neutral protease II in heat-induced soy sauce sediment formation
Source: Appl Microbiol Biotechnol. 2026 Jul 3;110:285. doi: 10.1007/s00253-026-13923-w (PMC13601390; doi:10.1007/s00253-026-13923-w)
Supplement: Supplementary file 2 [file 253_2026_13923_MOESM2_ESM.pdf]

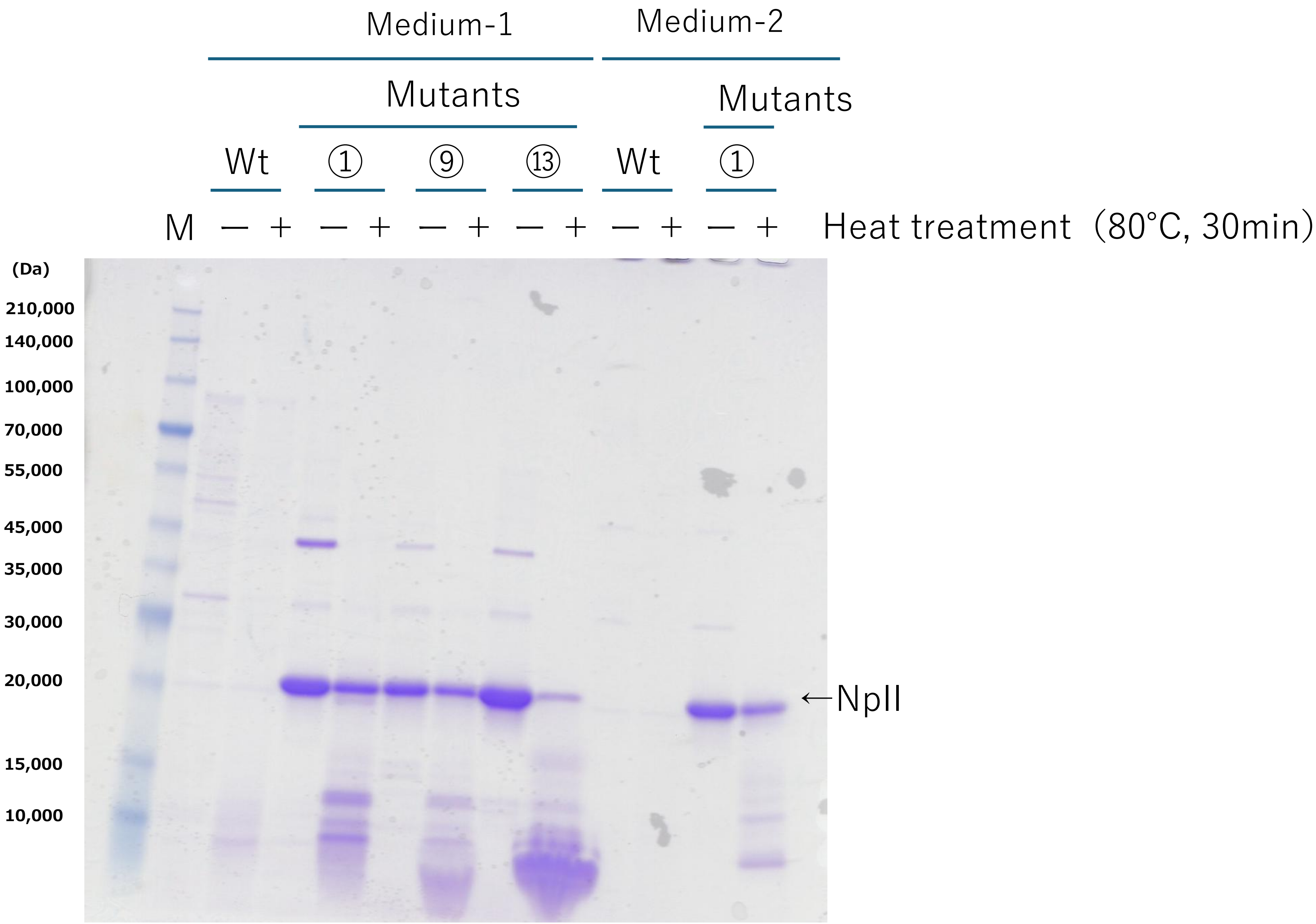

“The untrimmed SDS–PAGE image for Fig. S6 is provided.”
